# Identification and Characterization of Genes Associated With Wood Property Traits via Genome Sequencing in Fast‐Growing Species of Genus *Neolamarckia*

**DOI:** 10.1111/eva.70314

**Published:** 2026-08-24

**Authors:** Chao Wu, Wan‐Xin Chen, Zi‐Han He, Kun‐Xi OuYang, Qin‐Ming Que, Hui Xie, Wen‐Xiang Xu, Shiou Yih Lee, Siti Halimah Larekeng, Shi Shi, Xin‐Sheng Hu

**Affiliations:** ^1^ College of Forestry and Landscape Architecture South China Agricultural University Guangzhou China; ^2^ Guangdong Key Laboratory for Innovative Development and Utilization of Forest Plant Germplasm Guangzhou China; ^3^ Faculty of Health and Life Sciences INTI International University Nilai Malaysia; ^4^ Faculty of Health Science Shinawatra University Pathum Thani Thailand; ^5^ Forestry Engineering Study Program Hasanuddin University Makassar Indonesia

**Keywords:** GWAS, lignin biosynthesis, *N. cadamba*, *Neolamarckia macrophylla*, wood property traits

## Abstract

The *Neolamarckia* (Rubiaceae) genus is composed of only two species: *N. cadamba* and *N. macrophylla* . Both species are fast‐growing plants with significant industrial and medicinal values. Here, we provided a high‐quality chromosome‐level assembly of the *N. macrophylla* genome for the first time. The assembled genome size was approximately 598.43 Mb, with contig N50 and scaffold N50 lengths of 23.41 and 24.54 Mb, respectively. The two species diverged at 6.5–23.5 Mya and one whole‐genome duplication (WGD) event was detected to occur prior to their divergence. The assembled genomes showed that *N. macrophylla* had fewer protein‐coding genes (34,411 vs. 35,461) but substantially more expanded gene families than *N. cadamba* . Most expanded gene families were under purifying selection in both *N. macrophylla* and *N. cadamba* . We identified genes associated with seven wood property traits of *N. cadamba* through GWAS based on resequencing datasets of 204 trees in total collected from a provenance trial, with 100 newly resequenced trees at age 12 and 104 previously resequenced trees at age 7. Almost all identified candidate genes were under purifying selection. Further analysis indicated that genes encoding 11 key enzymes in the lignin biosynthesis pathway were under purifying selection between *N. cadamba* and *N. macrophylla* . The overall results imply that strong selection effectively impeded evolutionary divergence between the two species in genes associated with wood property traits. The study provides insights into genes regulating wood property traits of industrial values, and a useful genomic resource for developing molecular markers for breeding *Neolamarckia* varieties.

## 1 Introduction

The *Neolamarckia* genus of the Rubiaceae family is ditypic, containing only two species: *N. cadamba* (Roxb.) Bosser and *N. macrophylla* (Roxb.) Bosser. The *Neolamarckia* genus forms a monophyletic group (Shi et al. 2020; Dwiyanti et al. 2025). Their inflorescences are solitary and capitate, positioned terminally, and primarily pollinated by insects. *N. cadamba* is characterized by its leaves with distinct petioles and the upper part of the ovary, which has four distinct locules and four hollow cartilaginous structures (Ridsdale 1978). It is naturally distributed across regions with favorable hydrothermal conditions, including South China, Southeast Asia, South Asia, and North Australia (https://www.gbif.org/species/2896883, accessed 18 January 2026). It has a long history of domestication and cultivation in native regions and was successfully introduced to African and Central American countries for ornamental and timber purposes. In contrast, *N. macrophylla* is characterized by more or less sessile leaves; the upper part of its ovary is two‐loculed and has four small solid cartilaginous structures (Ridsdale 1978). It has a more restricted natural range, occurring solely on Sulawesi and Maluku Island, Indonesia, and is a threatened species. The two species are sympatric on Sulawesi and Maluku Island (Bosser 1984; Chen and Taylor 2011). Both species are pioneer species and thrive in fertile soils with high humidity (1200–2400 mm for annual precipitation) and high temperature (20°C–24°C for annual average temperature). *Neolamarckia* species have multiple significant values for industrial, ecological, and medicinal applications.

Two notable features make the *Neolamarckia* genus an important plant resource for industrial and medicinal applications. One is the use of the species as medicinal material (Pandey and Negi 2016; Qureshi et al. 2021; Balan et al. 2025). *N. cadamba* has been utilized in traditional medicine in Southeast and South Asian countries for a long time. Modern studies demonstrated that *N. cadamba* bark extracts were rich in cadambine, various flavonoids, alkaloids, and terpenoids, exhibiting significant medicinal effects on antioxidant, antidiarrheal, and antitumor activities. *N. cadamba* , the miracle and gem tree, was recognized as a highly important medicinal plant, representing a resource plant with substantial developmental potential and application value (Mojiol et al. 2014; Pandey and Negi 2018; Yadav et al. 2022; Singh et al. 2023).

The second feature is the combination of fast‐growing and slow‐aging characteristics (about 8 years for sexual maturity) where *N. cadamba* can attain 45 m in height and 1.6 m in trunk diameter (Mojiol et al. 2014). For instance, the tree exhibits an annual increase of 2–3 m in height from 2 to 5 years after planting, and an annual increase of 3.0–4.5 cm in diameter at breast height (DBH) from 3 to 7 years. The tree can attain 25 m in height and 55 cm in DBH after the 14th year of planting (Su et al. 2007). *Neolamarckia* species are large trees characterized by straight trunks and opposite leaves, and are valued for their high‐quality timber, which is easily worked and utilized for furniture, architectural decoration, and paper pulp products (Cahyono et al. 2016; Pandey and Negi 2016). Their wood properties are characterized by medium fiber length (approximately 1.43 mm), with the fiber width, cell wall thickness, and stiffness coefficient comparable to those of softwood plants, such as *Pinus kesiya* and *Picea abies* . The low lignin and high total cellulose contents enable the species to yield higher pulp production under milder heating conditions (Lal et al. 2010).

Since fast‐growing tree species can rapidly accumulate biomass within a short time, this is frequently accompanied by trade‐offs in wood properties. The growth–wood quality trade‐off is largely mediated by changes in cellular architecture and cell wall deposition, whereby rapid cambial activity and shortened secondary wall formation often yield lower wood density, alter fiber morphology, and enlarge vessel elements. Such anatomical shifts can enhance hydraulic efficiency and growth performance but may compromise mechanical strength, durability, and industrial utilization, including pulp and paper quality (Climent et al. 2024; Zhu and Li 2024). Accordingly, here we concentrated on a few representative traits—fiber length, fiber diameter, vessel length, vessel diameter, basic density, fiber length‐to‐diameter ratio, and vessel length‐to‐diameter ratio, which collectively capture the characteristics in cell structure, mechanical performance, and utilization‐related properties of wood and examined the genetic basis of these wood property traits. In addition, as the core component in vascular plant cell walls, lignin enhances plant mechanical support, enables water transport and confers resistance against biotic and abiotic stresses (Boerjan et al. 2003). Moreover, recent studies have revealed that lignin biosynthesis is also influenced by noncoding RNAs (Wang et al. 2020; Zhao et al. 2025). Variation in lignin content could affect wood properties (Friedman and Cook 2000; Arisandi et al. 2025). Since both species are fast‐growing but potentially different in wood property traits (Wu et al. 2026), it is interesting to examine the evolutionary mechanisms that generate differences in wood properties. Given the well‐known pathways for different types of lignin biosynthesis (Hu et al. 1999; Boerjan et al. 2003), we examined the evolutionary divergence between the two species in genes controlling lignin biosynthesis.

The purpose of this study is to identify candidate genes associated with wood property traits of *N. cadamba* and characterize the evolutionary divergences between the two species in the candidate genes and the key genes for lignin biosynthesis. Previous studies identified a few genes associated with xylogenesis and lignin biosynthesis (Ho et al. 2014; Yiing et al. 2014; Pang et al. 2015) and the secondary cell wall biosynthesis in *N. cadamba* (Ouyang et al. 2016). Few studies have been conducted on *N. macrophylla* . Here, we conduct a genome‐wide association study (GWAS) based on resequencing of 204 individuals collected from a progeny trial covering 11 provenances across the full natural distribution range of *N. cadamba* in China and 1 provenance from Indonesia. Seven wood property traits were investigated in two samples that were collected with different sampling schemes (one at age 7 and the other at age 12).

To characterize evolutionary divergence between the two species in genes controlling wood property traits, we conducted a chromosome‐level genome assembly of *N. macrophylla* (2*n* = 44 chromosomes) using both Illumina and PacBio platforms for the first time. A high‐throughput chromosome conformation capture (Hi‐C) map was produced to anchor the assembled contigs on 22 pseudochromosomes. Comparative genomic analyses were conducted to assess genomic divergence between *N. cadamba* and *N. macrophylla* , including estimation of their species divergence times, the WGD and expansion/contraction of gene families. Natural selection on both identified candidate genes and the key genes for lignin biosynthesis was then examined in terms of the ratio (*K*_a_/*K*_s_) of nonsynonymous substitution rate (*K*_a_) to synonymous substitution rate (*K*_s_). The overall study revealed the interspecific divergence of genes associated with wood property traits and would provide useful references for molecular breeding and genetic conservation in the *Neolamarckia* genus.

## 2 Materials and Methods

### 2.1 Genome Sequencing and Assembly of *N. macrophylla*

#### 2.1.1 Plant Materials and DNA Extraction

Fresh leaves were collected from an ex situ *N. macrophylla* tree planted at the Herb Garden of INTI International University, which originated from a plantation in Kuala Muda District, Kedah State of Malaysia (Geographic coordinates: 100°28′49.95″ E, 5°47′44.72″ N) (Figure 2A). The sampled tree was identified by SYL, SHL, and SS. Voucher specimens were deposited in both the Biotechnology Laboratory at INTI International University and the Herbarium of South China Agricultural University (CANT) (Accession number: 32221). Genomic DNA was extracted using QIAGEN Genomic‐tips at the Biotechnology Laboratory of INTI International University by following standard protocols. The DNA sample was then entrusted to Biomarker Technologies Co. Ltd. (Beijing, China).

#### 2.1.2 Genome Sequencing and Assembly

Genome sequencing was performed with a multi‐platform strategy: Illumina‐based sequencing, Hi‐C sequencing, and PacBio sequencing. For genome survey, genomic DNA was used to construct a 350‐bp library and sequenced on the Illumina NovaSeq 6000 platform. The k‐mers were counted with Jellyfish v2.1.4 (Marçais and Kingsford 2011), and the genomic characteristics (genome size, repeat content, and heterozygosity rate) were estimated using Genomescope v2.00 (Ranallo‐Benavidez et al. 2020) with parameters ‐k 21 ‐p 2 ‐m 100,000. The Hi‐C library was sequenced on the Illumina X‐PIUS platform using the Sequencing by Synthesis (SBS) technology. PacBio sequencing was performed using the Circular Consensus Sequencing (CCS) mode on the PacBio Revio platform, generating High Fidelity (HiFi) reads. The details of library construction and quality control are provided in the Supporting Information.

The genome assembly was conducted as follows: PacBio HiFi clean reads were assembled into contigs using Hifiasm v0.19 with parameters ‐‐I 2 ‐n 4 (Cheng et al. 2021). Hi‐C reads were mapped to the contigs using bwa v0.7.17‐r1188 with default parameters (Li and Durbin 2009). The invalid read pairs were filtered with HiC‐Pro v2.10.0 (Servant et al. 2015). Then, Hi‐C data were employed to assist genome assembly. First, a Hi‐C based error correction was performed: the contig‐level genome was fragmented into 50‐kb pieces, and Hi‐C data were used to reassemble these pieces; positions that could not be restored to the original assembly sequences were considered candidate error regions. Within these regions, sites with low Hi‐C coverage depth were identified as error points, thereby completing error correction of the preliminary genome assembly. The error‐corrected genome was then assembled using LACHESIS v2.0.1 (Burton et al. 2013) with parameters CLUSTER_MIN_RE_SITES = 92, CLUSTER_MAX_LINK_DENSITY = 2, ORDER_MIN_N_RES_IN_TRUNK = 12, and ORDER_MIN_N_RES_IN_SHREDS = 10. Subsequently, manual adjustment and inspection based on chromosomal interaction intensity were performed, ultimately yielding a chromosome‐level genome assembly.

The completeness of the genome assembly was assessed using two approaches: Core Eukaryotic Genes Mapping Approach v2.5 (CEGMA) (Parra et al. 2007) and Benchmarking Universal Single‐Copy Orthologs v5.2.1 (BUSCO) (Simão et al. 2015). CEGMA employed a reference core gene set comprising 458 highly conserved eukaryotic genes, and identified conserved orthologous regions within the assembly through a combination of TBLASTN (Gertz et al. 2006), GeneWise v2.4.1 (Birney et al. 2004), and Geneid (Parra et al. 2000), thereby evaluating the assembly completeness at the gene content level. BUSCO v5.2.1 was run against the embryophyta lineage dataset from OrthoDB 10 (http://cegg.unige.ch/orthodb). In addition, to evaluate the uniformity of sequencing coverage and the integrity of the assembled sequences, two independent read‐mapping strategies were applied. Illumina paired‐end short reads were aligned to the assembly using BWA v0.7.17‐r1188, and PacBio HiFi reads were mapped using Minimap2 (Li 2018). For each dataset, the mapping rate, genome coverage, and depth distribution were computed.

#### 2.1.3 Genome Structure and Annotations

Repetitive sequences primarily consist of interspersed repeats and tandem repeats. The interspersed repeats are mainly transposable elements (TEs), for which two parallel de novo prediction strategies were employed.

RepeatModeler2 v2.0.1 (Flynn et al. 2020) was employed for de novo repeat prediction, and RepeatClassifier was subsequently used to classify the prediction results utilizing the known Dfam v3.5 database (Storer et al. 2021). Long terminal repeat retrotransposons (LTRs) were predicted by LTR_retriever v2.9.0 (Ou and Jiang 2018) based on de novo predictions using LTRharvest v1.5.10 (Ellinghaus 2008) and LTR_FINDER v1.07 (Xu and Wang 2007). These de novo predictions were then merged with the known database entries, followed by redundancy removal to generate a species‐specific repeat sequence database. Finally, RepeatMasker v4.1.2 (Tarailo‐Graovac and Chen 2009) was utilized to annotate transposable elements (TEs) within the genome based on this curated repeat database. Tandem repeats were primarily predicted using the MicroSatellite identification tool (MISA v2.1) (Beier et al. 2017) and Tandem Repeat Finder (TRF, version v409, with parameters: 2 7 7 80 10 50 500 ‐d ‐h) (Benson 1999).

Gene prediction was performed using three approaches. Ab initio prediction was conducted using Augustus v3.1.0 (Korf 2004) and SNAP (2006‐07‐28) (Stanke et al. 2008). Homology‐based prediction was carried out using GeMoMa v1.7 (Keilwagen et al. 2016). For second‐generation transcriptome‐based prediction, transcripts assembled via two distinct methods were utilized: one set was obtained using Hisat2 v2.1.0 (Kim 2015) and StringTie v2.1.4 (Pertea et al. 2015), followed by gene prediction with GeneMarkS‐T v5.1 (Tang et al. 2015); the other set was assembled de novo using Trinity v2.11 (Grabherr et al. 2013), and subsequently subjected to gene prediction with PASA v2.4.1 (Haas 2003). Third‐generation transcriptome reads were aligned using GMAP (2020‐06‐30) (Wu and Watanabe 2005), underwent splice site identification, and were then used for gene prediction with PASA v2.4.1 (Haas 2003). The RNA sequencing details are provided in the Supporting Information. Finally, the prediction results from these three approaches were integrated using EVidenceModeler (EVM v1.1.1) (Haas et al. 2008), and the integrated gene models were structurally refined using PASA v2.4.1.

Noncoding RNAs comprise a variety of functionally characterized RNAs. MicroRNAs (miRNAs), small nucleolar RNAs (snoRNAs), and small nuclear RNAs (snRNAs) were predicted based on the Rfam database v14.5 (Griffiths‐Jones et al. 2005) using Infernal v1.1 (Nawrocki and Eddy 2013). Ribosomal RNA (rRNA) prediction was primarily performed using barrnap v0.9 (Loman 2017). Transfer RNA (tRNA) genes were identified using tRNAscan‐SE v1.3.1 (Lowe and Eddy 1997).

Pseudogenes are defined as sequences that exhibit similarity to functional genes but have lost their original function owing to mutations (insertions and/or deletions). Homologous sequences (putative gene candidates) were identified by GenBlastA v1.0.4 (She et al. 2009) on the genome after masking the functional gene loci, and GeneWise v2.4.1 (Birney et al. 2004) was then used to detect premature stop codons and frameshift mutations within the sequences to predict pseudogenes. Motif annotation was performed using InterProScan v5.34‐73.0 (Jones et al. 2014).

The predicted gene sequences were comprehensively annotated against the following databases: the NCBI nonredundant protein database (NR; https://www.ncbi.nlm.nih.gov/refseq/about/nonredundantproteins/), eggNOG (http://eggnog5.embl.de/#/app/home; Huerta‐Cepas et al. 2019), Gene Ontology (GO; https://www.geneontology.org/), the Kyoto Encyclopedia of Genes and Genomes (KEGG; Kanehisa et al. 2016), TrEMBL (https://www.uniprot.org/help/uniprotkb), eukaryotic Clusters of Orthologous Groups (KOG; https://mycocosm.jgi.doe.gov/help/kogbrowser.jsf), SWISS‐PROT (http://www.uniprot.org/; Boeckmann et al. 2003), and Pfam (http://pfam.xfam.org/; Finn et al. 2006).

### 2.2 Comparative Genomic Analysis

To assess the divergence between *N. cadamba* and *N. macrophylla* , orthologous groups were inferred from the protein sequences of nine species (seven other plant species: *Arabidopsis thaliana* , *Amborella trichopoda*, *Coffea canephora* , *Cycas panzhihuaensis* , *Eucalyptus grandis* , *Ginkgo biloba* , *Toona ciliata* ) using OrthoFinder v2.4 (Emms and Kelly 2019) with DIAMOND (Buchfink et al. 2015) alignments (*E*‐value threshold: 10^−3^). The resulting gene families were functionally annotated with the PANTHER v15.0 database (Mi et al. 2019). Finally, GO and KEGG pathway enrichment analyses were performed on species‐specific gene families.

A phylogenetic tree was constructed using IQ‐TREE v1.6.11 (Nguyen et al. 2015) based on single‐copy gene sequences. Sequences of each single‐copy gene family were aligned using MAFFT v7.205 (Katoh et al. 2009) with parameters ‐‐localpair ‐‐maxiterate 1000. Poorly aligned or highly divergent regions were subsequently removed using Gblocks v0.91b with parameters ‐‐b5 h (Talavera and Castresana 2007). The aligned sequences for each species were then concatenated to generate a supergene matrix. ModelFinder, integrated within IQ‐TREE, was employed to identify the best‐fit substitution model. Using this optimal model, a maximum likelihood (ML) tree was inferred with 1000 bootstrap replicates. We used *G. biloba* and *C. acuminata* (formerly *C. panzhihuaensis* ) as outgroups to root the tree. We selected six other plants for comparison, including one model plant *A. thaliana* (Arabidopsis Genome Initiative 2000), one gymnosperm species *C. revoluta* , and four angiosperm species ( *A. trichopoda* , *T. ciliata* , *E. grandis* , and *C. canephora* ). Divergence times were estimated using MCMCTREE within the PAML v4.9i package (Yang 2007). Fossil calibration points obtained from TimeTree (http://www.timetree.org/) were applied: *N. macrophylla* versus *G. biloba* (326.4–336.8 Mya), *N. macrophylla* versus *A. trichopoda* (179.9–204.9 Mya), and *N. cadamba* versus *C. canephora* (55.4–72.2 Mya). These calibrations were used to constrain the time estimates derived from the computational algorithm. The gradient and Hessian required for divergence time estimation in MCMCTREE were computed. Finally, divergence times were estimated under a correlated molecular clock model using the JC69 substitution model with maximum likelihood. Two independent runs were performed to assess consistency, with MCMC parameters set as follows: burnin = 500,000, sampfreq = 30, and nsample = 15,000,000.

Gene family evolution was analyzed using CAFE v4.2 (Han et al. 2013). This software employed the time‐calibrated phylogenetic tree and the gene family clustering results to estimate the ancestral gene family size at each branch under a birth‐death model. Consequently, gene family contraction and expansion events relative to the inferred ancestors were predicted for the focal species. A gene family was defined as having undergone significant expansion or contraction only if both its family‐wide *p* value and Viterbi *p* value were < 0.05.

Collinearity analysis was performed using DIAMOND v0.9.29.130 (Buchfink et al. 2015) to align gene sequences between the two species, identifying homologous gene pairs (*E*‐value < 10^−5^, *C*‐score > 0.5). Putative syntenic blocks were subsequently identified using MCScanX (Wang et al. 2012) with parameter ‐m 15, which assessed chromosomal collinearity by determining physical proximity of homologous pairs within their respective genomes based on GFF3 annotations.

WGD was detected using synonymous substitution rate (*K*_s_) analysis and the fourfold synonymous third‐codon transversion (4DTv) method. The latter quantifies transversion mutations at fourfold degenerate sites (third‐codon positions where any nucleotide substitution encodes the same amino acid). Analyses were conducted using wgd v1.1.1 (Zwaenepoel and Van de Peer 2019). For 4DTv calculation, custom scripts (https://github.com/JinfengChen/Scripts) were employed to compute the proportion of transversion substitutions at fourfold degenerate sites within homologous gene pairs, followed by correction using the HKY substitution model.

Identification of LTR‐RT sequences was based on Subsection 2.1.3. For each identified LTR‐RT, the paired LTR sequences were extracted and aligned using MAFFT with parameters ‐‐localpair ‐‐maxiterate 1000. The synonymous substitution rate (*K*_s_) between the aligned LTR pairs was then calculated using the distmat program within EMBOSS v6.6.0 (Rice et al. 2000). Divergence time (*T*) was estimated using the formula *T* = *K*_s_/(2 × *r*), where the substitution rate (*r*) was set to 7 × 10^−9^ substitutions per site per year. Statistical comparisons of LTR insertion time distributions were performed using the Kolmogorov–Smirnov (K‐S) test implemented in R v4.4.3. (R Core Team 2025).

To assess evolutionary mechanisms underlying expanded genes in *N. macrophylla* and *N. cadamba* , the KaKs_Calculator 3.0 (Zhang 2022) was used to estimate the ratio of nonsynonymous (*K*_a_) to synonymous (*K*_s_) substitution rates, *K*_a_/*K*_s_. The identified orthologous genes between the two species were used to calculate *K*_a_/*K*_s_ and to evaluate if these genes were under positive (*K*_a_/*K*_s_ > 1) or purifying (*K*_a_/*K*_s_ < 1) selection, or under neutrality (*K*_a_/*K*_s_ = 1).

### 2.3 Procedures for GWAS of *N. cadamba*

#### 2.3.1 Plant Materials and Phenotyping of Wood Property Traits

**FIGURE 1:**
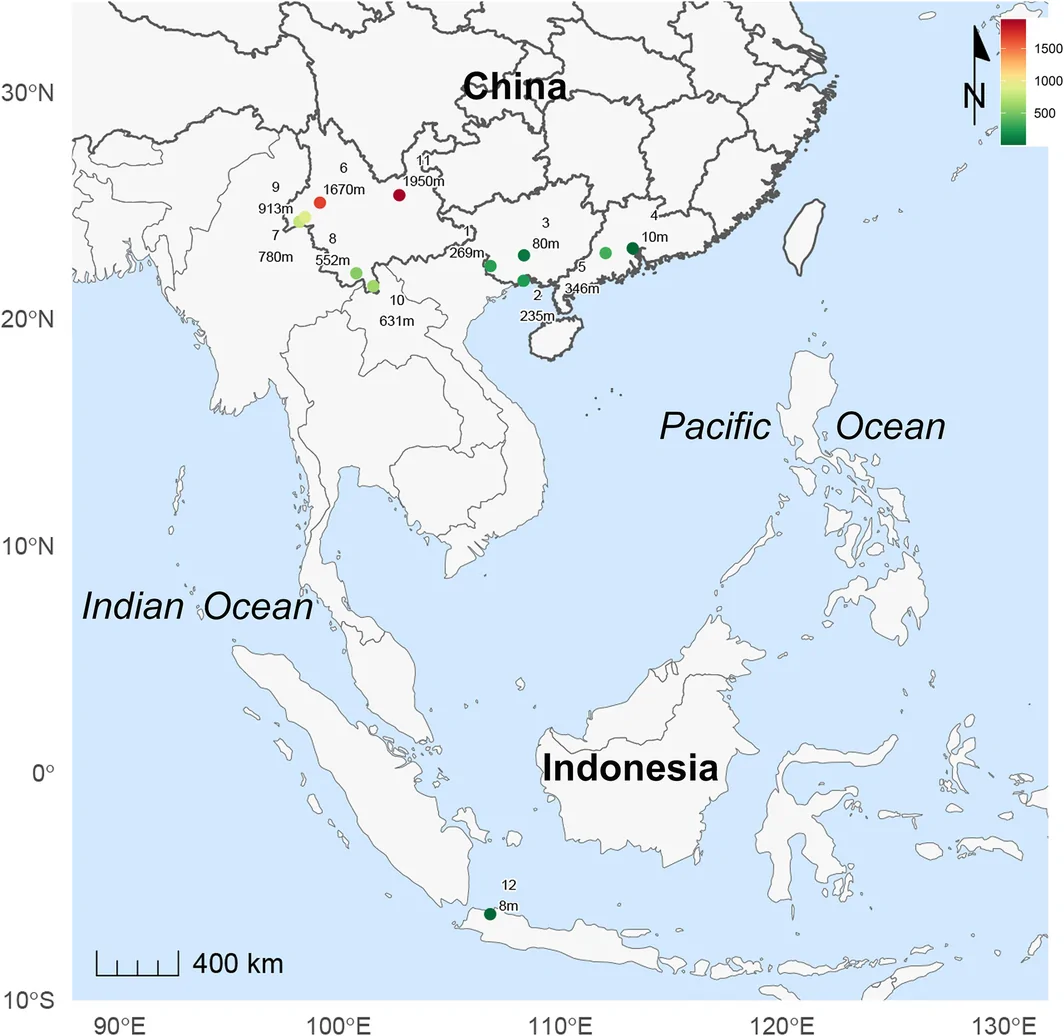
The localities of 12 provenances where seeds were collected for a provenance trial of *N. cadamba* . The progeny test was set up in 2014 at Jijia Town, Leizhou City, Guangdong Province. The numbers from 1 to 12 localities in the map indicate sampling sites of Longzhou, Fangchenggang, Nanning, Guangzhou, Yunfu, Baoshan, Dehong, Jinghong, Mangshi, Mengla, Kunming, and Indonesia, respectively.

The samples for GWAS were collected from a provenance progeny trial established at the forest farm in Jijia Town, Leizhou City, Guangdong Province (21°10′06″ N, 110°21′34″ E) in 2014. The climate belongs to a tropical monsoon climate, with the average annual sunshine duration of 2031.5 h (approximately 169 days of sunshine), the average annual temperature of 23.6°C, and the average annual precipitation of 1671.5 mm, based on the 30‐year average (1991–2020) (http://gd.cma.gov.cn/zjsqxj/zwgk_2986/gggs_2995/202604/P020260416559526278968, accessed 30 June 2026). This provenance trial of *N. cadamba* was composed of 11 provenances covering its range‐wide distribution in China and 1 Indonesian population (Figure 1), with 60 half‐sib families in total (Table 1). The map was generated in R v4.4.3 using the ggplot2 package (Wickham 2016), with spatial boundaries sourced from the rnaturalearth package (https://CRAN.R‐project.org/package=rnaturalearth). The experiment was set up using a randomized complete block design, with 11 blocks, 5 individuals per plot, and the planting space of 3 m × 3 m. Two samples were separately collected for resequencing and phenotyping of wood property traits. The first sample (104 trees) was collected at the 7th year after planting, and individuals with large variations in height were selected within the randomly chosen families of a provenance. The resequencing data of 104 individuals at age 7 were downloaded from the NCBI BioSample database under accession number SAMN15700859, as reported by Zhao et al. (2022). The second sample (100 trees) was collected at age 12, where individuals were randomly selected within randomly chosen families.

**TABLE 1:** Samples for genome resequencing from 12 provenances of *N. cadamba* at ages 7 (the first sample) and 12 (the second sample).

Provenances	Longitude (°E)	Latitude (°N)	Elevation (m)	Total half‐sib families	Sample ID at age 7 (sample size within family)	Sample ID at age 12 (sample size within family)	Total sample size
Longzhou, Guangxi	106.86	22.35	269	3	D1‐D6 (6)	C15, C105, C115 (3)	9
Fangchenggang, Guangxi	108.35	21.70	235	1	D7, D8 (2)	C11 (1)	3
Nanning, Guangxi	108.37	22.82	80	5	D9‐D18 (10)	C14, C46, C106, C117 (4)	14
Guangzhou, Guangdong	113.27	23.13	10	3	D19, D21‐D24 (5)	C20, C29 (2)	7
Yunfu, Guangdong	112.05	22.92	346	1	D26 (1)	C47 (1)	2
Baoshan, Yunnan	99.17	25.14	1670	6	D27‐D36 (10)	C1, C2, C12, C22, C27, C35, C60, C82 (8)	18
Dehong, Yunnan	98.61	24.44	780	10	D37‐D46 (10)	C16, C17, C26, C30, C31, C33, C34, C38, C39, C50, C75, C78, C83, C84, C85, C87, C88, C107, C109, C110 (20)	30
Jinghong, Yunnan	100.81	22.03	552	21	D47‐D65, D67‐D88 (40)	C3–C10, C21, C25, C36, C37, C41, C42, C44, C45, C48, C49, C51–C58, C61–C72, C74, C76, C77, C79–C81, C101, 102, 103, C104, C111, C113, C116 (51)	91
Mangshi, Yunnan	98.59	24.43	913	2	D89‐D92 (4)	C23, C24 (2)	6
Mengla, Yunnan	101.57	21.46	631	4	D93‐D100 (8)	C13, C32, C40, C59 (4)	12
Kunming, Yunnan	102.75	25.47	1950	3	D103‐D108 (6)	C108, C112, C114 (3)	9
Indonesia	106.85	−6.21	8	1	D101, D102 (2)	C19 (1)	3

The phenotypic traits for all sampled trees were the diameter at breast height (DBH) and five wood property traits that were the fiber length (FL), fiber diameter (FD), vessel length (VL), vessel diameter (VD), and basic density (BD). Two additional traits were the fiber aspect ratio (FL/FD) and vessel aspect ratio (VL/VD). DBH was measured at 1.3 m above ground level using a diameter tape, and this trait was not focused on here. The method of Chen and Xie (2003) was used to measure wood property traits. Wood cores were extracted at 1.3 m above ground level in a north–south direction using an increment borer (5 mm internal diameter), ensuring the borer penetrated through the pith. Cores were immediately placed in sealed bags, labeled, and transported to the laboratory for wood property assessment. For fiber and vessel length characterization, the wood core samples (approximately 2 mm in diameter and 30–50 mm in length) were placed in test tubes and treated with 10–20 times their volume of a chromic acid‐nitric acid maceration fluid. The tubes were heated over an alcohol lamp at 96°C–98°C for 5–8 min. A subsample was transferred to a glass slide, gently pressed with forceps, and examined under a microscope to ensure complete maceration. After complete maceration, the samples were thoroughly rinsed with distilled water to remove the maceration fluid, stained with a small volume of 0.5% safranin aqueous solution, and gently teased apart using a glass rod. The lengths of fibers and vessels per sample were measured under a Leica DM1000 LED optical microscope, and the measurements were performed using the Leica Application Suite X software (v3.4.1.17822). For measuring fiber diameter (FD) and vessel diameter (VD), small wood blocks (approximately 1 mm × 1 mm × 1 mm) were prepared, immersed in a beaker containing water for softening, and then sectioned transversely at a thickness of 20–25 μm using a microtome (YAMATO REM 710). The sections were stained with safranin, dehydrated through a graded ethanol series, cleared in xylene, and permanently mounted with neutral resin mounting medium. The prepared slides were observed under a Leica DM1000 LED microscope, and transverse‐section images were captured. Using the Leica Application Suite X software (v3.4.1.17822), intact fiber cells and vessels were randomly selected from each cross‐section, and their tangential diameters were measured. Under strict quality control with fragmented and irregular fibers and vessels excluded, 30 effective replicates of fibers and vessels per sample were measured for FL, FD, VL, and VD in the first sample (age 7). Nineteen effective replicates per sample were measured for FL, VL, and VD, and 10 effective replicates per sample were measured for FD in the second sample (age 12). For the basic density determination, specimens were prepared according to China National Standard GB/T 1928–2009. Specimen surfaces (approximately 5 mm × 5 mm × 5 mm) were planed smoothly, with two opposing edges aligned approximately parallel to the growth rings and the other two edges perpendicular to them. Specimens exhibiting visible defects were excluded. Each specimen was numbered, saturated in water, and its volume (*V*) was determined by water displacement. The specimens were then oven‐dried to a constant weight to obtain their oven‐dry mass (*M*). The basic density (*ρ*, g/cm^3^) was calculated as *ρ* = *M*/*V*.

#### 2.3.2 Evaluation of Phenotypic Variation

The coefficients of variation (CV = SD/mean) for the seven wood property traits were calculated using Excel 2019 (Microsoft, USA). The results were visualized using Origin v2021 (OriginLab, USA). Pearson correlation matrix among wood property traits was calculated using the cor() function and visualized as heatmaps using the ggplot2 package (Wickham 2016) in R v4.4.3. Statistical significance was tested using the Hmisc package (https://CRAN.R‐project.org/package=Hmisc) for all variable pairs in R v4.4.3. Shapiro–Wilk normality test was performed for each wood property trait in R 4.4.3. Principal component analysis (PCA) was conducted using the prcomp function in R, with the data centrally scaled (scale. = TRUE) to eliminate impacts of different measurement scales and units among traits. Seven phenotypic traits were used to calculate PCA. The first two principal components (PC1 and PC2), with the first and second largest eigenvalues, were used to evaluate variation of all samples. PCA plot was visualized using the fviz_pca_ind function from the factoextra package (https://CRAN.R‐project.org/package=factoextra).

#### 2.3.3 Resequencing and SNP Callings

Fresh leaves from 100 trees at age 12 were collected (Table 1 for details) and entrusted to Novogene Bioinformatic Company, Beijing, for DNA extraction and genome resequencing. The experimental procedure followed the standard protocol provided by Illumina, including DNA sample quality check, library construction, library quality testing, and library sequencing. These details are provided in the Supporting Information.

Quality‐approved sequencing data were aligned to the *N. cadamba* reference genome (https://figshare.com/s/ed20e0e82a4e7474396b) using BWA‐MEM (Li and Durbin 2009) with parameters mem ‐t 4 ‐k 32 ‐M. The resulting alignments were processed with SAMtools (Li, Handsaker, et al. 2009) to remove duplicate reads (rmdup command). Single nucleotide polymorphisms (SNPs) were called per individual using SAMtools mpileup (Li, Handsaker, et al. 2009) with parameters ‐m 2 ‐F 0.002 ‐q 1 ‐C 50 ‐t SP ‐t DP ‐t AD. To minimize false positives, SNP calls were filtered according to the following criteria: (1) The read depth supporting the variant allele was ≥ 4 and ≤ 10,000,000; (2) The mapping quality (MQ) was ≥ 20.

#### 2.3.4 Population Genomic Analysis, GWAS, and Gene Annotations

VCFtools (Danecek et al. 2011) was used to perform variant quality control. Only biallelic SNPs with minor allele frequency (MAF) ≥ 0.05 and missing rate ≤ 10% were retained for downstream analyses. The genome‐wide distribution of SNP density was visualized by R package CMplot v4.5.1 (https://github.com/YinLiLin/CMplot), and the SNP density was calculated based on the 1‐Mb window size. Hardy–Weinberg equilibrium (HWE) was tested for each SNP using VCFtools with the ‐‐hardy parameter. The distribution of *p* values was then visualized by ggplot2 in R v4.4.3. The genome‐wide nucleotide diversity (*π*) was calculated with VCFtools, with a window size of 10 kb and a step size of 1 kb (‐‐window‐pi 10,000, ‐‐window‐pi‐step 1000). The density distribution of *π* values was visualized using ggplot2 in R v4.4.3. Linkage disequilibrium (LD) decay between SNPs was calculated using PopLDdecay (v1.90) (Zhang et al. 2019), and visualized by ggplot2 in R v4.4.3.

Loci under Hardy–Weinberg disequilibrium (*p* < 10^−6^) were removed using VCFtools. The quality‐controlled VCF file was converted into PLINK binary files (BED/BIM/FAM) using PLINK v1.9 (Purcell et al. 2007) for subsequent GWAS analyses. A relatedness matrix was estimated using GEMMA (‐gk 1), and association tests were conducted using a linear mixed model with the Wald test (‐lmm 1) (Zhou and Stephens 2012). The significance threshold was calculated using the Genetic Type I error calculator (GEC) (Li et al. 2012). Manhattan and quantile‐quantile (Q‐Q) plots were visualized using the R package CMplot v4.5.1. We identified candidate genes within 50‐kb flanking regions of significant SNPs based on LD decaying patterns. Functional annotations of these genes were referred to those derived from genome assembly mentioned in Subsection 2.1.3.

### 2.4 Test of Natural Selection on Genes Associated With Wood Property Traits

To assess evolutionary mechanisms underlying genes associated with wood property traits, we estimated the ratio of nonsynonymous (*K*_a_) to synonymous (*K*_s_) substitution rates, *K*_a_/*K*_s_, using KaKs_Calculator 3.0 (Zhang 2022). Besides, based on the recognized pathways for different types of lignin biosynthesis (Bai et al. 2022), we estimated *K*_a_/*K*_s_ values of some key gene families. With a reference to gene sequences from *A. thaliana* via the TAIR database (https://www.arabidopsis.org/), candidate genes in *N. cadamba* and *N. macrophylla* were identified through two complementary approaches: (1) local BLAST searches against species‐specific protein databases using BioEdit v7.7.1 (*E*‐value < 0.01) (https://thalljiscience.github.io/page2.html), and (2) HMMER v3.0 (http://hmmer.org/) based scans of the same databases. Candidate sequences from both methods were merged and de‐duplicated. Conserved domains were subsequently annotated using NCBI Batch CD‐Search (https://www.ncbi.nlm.nih.gov/Structure/bwrpsb/bwrpsb.cgi), and genes exhibiting incomplete structural domains were eliminated to finalize the candidate gene sets related to lignin biosynthesis.

## 3 Results

### 3.1 Genome Sequencing, Assembly, and Annotation of *N. macrophylla*

**FIGURE 2:**
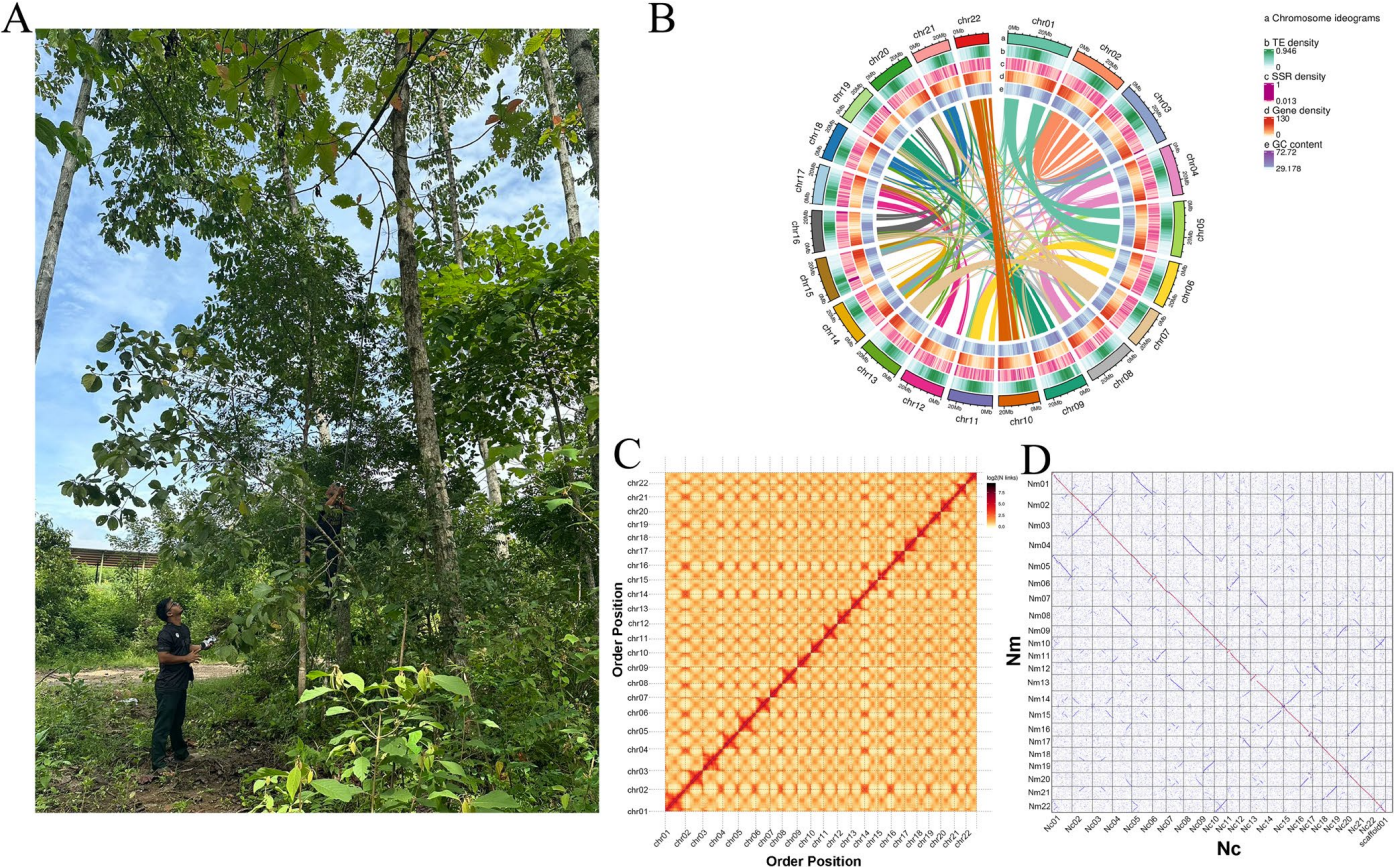
Morphological characteristic and genome assembly of *N. macrophylla* . (A) The tree of *N. macrophylla* on site was used for genome sequencing; (B) circular map of genomes; (C) chromosomal interaction heatmap of the Hi‐C assembly; (D) a dot plot for chromosome comparison.

To achieve a higher‐quality genome sequencing and assembly for *N. macrophylla* (2*n* = 2*x* = 44), a genome survey was performed. Analysis based on the k‐mer distribution profile (Figure S1) indicated an estimated genome size of approximately 556.38 Mb, with repetitive sequences accounting for 36.66%. The estimated genomic heterozygosity was 0.48%, indicating that it is a relatively simple genome. PacBio platform‐based HiFi sequencing generated approximately 37.33 Gb of clean data, with the total sequencing depth of approximately 67× (Table S1), and Hi‐C sequencing yielded approximately 79.04 Gb of clean data, with the total sequencing depth of approximately 139× (Table S2). Using the combined HiFi and Hi‐C sequencing data, the genome was anchored to 22 chromosomes (Table S3). Following Hi‐C‐based error correction, chromosome anchoring, and redundancy removal, the final *N. macrophylla* genome assembly was generated. This assembly had a total length of approximately 598.43 Mb, with a Contig N50 of 23.41 Mb and a Scaffold N50 of 24.54 Mb (Figure 2B, Table 2).

**TABLE 2:** The assembly and annotation of *N. macrophylla* genome.

	Number	Size (bp)
Genome assembly		
Total number of contigs	218	—
Contig N50	—	23,405,752
Contig N90	—	5,238,098
Maximum length of contigs (Mb)	—	35,559,994
Total number of scaffolds	210	—
Scaffold N50	24,540,723	—
Scaffold N90	20,818,997	—
Maximum length of scaffolds	—	36,004,532
GC	—	36.63%
Total numbers of gap	8	—
Genome length	—	598,429,791
Genome annotation		
Transposable elements	40.8%	244,161,058
Predicted protein‐coding genes	34,411	—
Noncoding RNA prediction	4843	—
Total number of pseudogene	37	—
The proportion of gene function annotation	97.7%	—
Regulatory motif annotation	1484	—
Average exon length (bp)	—	1676.50
Average intron length (bp)	—	2536.68

To evaluate the assembly quality of the *N. macrophylla* genome, the genome was partitioned into 300,000 bp bins (Figure 2C). The interaction strength signal between any two bins was defined as the number of Hi‐C read pairs spanning them. This analysis clearly distinguished 22 chromosomal groupings. Within each group, the interaction strength was higher along the diagonal than in off‐diagonal positions, indicating stronger interactions between adjacent sequences compared to distant sequences in the Hi‐C‐assisted chromosome assembly and showing high assembly quality (Figure 2D). Furthermore, CEGMA assessment revealed a completeness of 95.85%, while BUSCO evaluation indicated a completeness of 94.80%. The second‐generation (Illumina) read mapping yielded a mapping rate of 98.59%, coverage of 99.76%, and an average sequencing depth of 86×. The third‐generation (PacBio) read mapping resulted in a mapping rate of 98.96%, coverage of 99.99%, and an average depth of 63×. Collectively, these results demonstrated that the *N. macrophylla* genome assembly was highly accurate and complete.

After genome assembly, repetitive sequences were annotated. Interspersed repeats were predominantly transposable elements (TEs), yielding approximately 244.16 Mb of TE sequences and accounting for 40.80% of the genome size (Table 3). Tandem repeats were estimated to be approximately 21.46 Mb, representing 3.59% of the genome.

**TABLE 3:** Classification and content of repetitive elements (REs) identified in the genome.

Type	Number	Length	Rate (%)
ClassI:Retroelement	240,701	206,848,255	34.57
ClassI/LINE	17,487	6,870,787	1.15
ClassI/LTR/Caulimovirus	1641	2,534,188	0.42
ClassI/LTR/Copia	47,868	49,179,458	8.22
ClassI/LTR/ERV	3053	208,898	0.03
ClassI/LTR/Gypsy	77,337	100,012,213	16.71
ClassI/LTR/Ngaro	347	25,211	0
ClassI/LTR/Pao	128	8427	0
ClassI/LTR/Unknown	88,727	47,548,029	7.95
ClassI/SINE	4113	461,044	0.08
ClassII:DNA transposon	128,004	37,310,484	6.23
ClassII/Academ	5	295	0
ClassII/CACTA	2087	132,729	0.02
ClassII/Crypton	35	1912	0
ClassII/Dada	265	13,701	0
ClassII/Ginger	37	2347	0
ClassII/Helitron	45,602	15,144,328	2.53
ClassII/IS3EU	168	11,974	0
ClassII/Kolobok	385	36,480	0.01
ClassII/Maverick	137	9211	0
ClassII/Merlin	202	11,673	0
ClassII/Mutator	1969	1,377,154	0.23
ClassII/P	110	7192	0
ClassII/PIF‐Harbinger	592	36,000	0.01
ClassII/PiggyBac	63	2912	0
ClassII/Tc1‐Mariner	271	16,063	0
ClassII/Unknown	73,781	20,363,023	3.40
ClassII/Zisupton	118	6396	0
ClassII/hAT	2177	137,094	0.02
Unknown	35	2319	0
Total	368,740	244,161,058	40.80

Protein‐coding genes (PCGs) were predicted using a combination of homology‐based, ab initio, and transcriptome‐supported approaches, resulting in 34,411 predicted genes. These genes had an average length of 4213.18 bp, contained an average of 5.3 exons per gene, and had an average coding sequence (CDS) length of 1341.97 bp. Noncoding RNAs were identified as comprising 1015 tRNAs, 3491 rRNAs, 58 miRNAs, 66 snRNAs, and 213 snoRNAs. Additionally, 37 pseudogenes were annotated, with a total length of 73,569 bp and an average length of 1988.35 bp. Annotation also revealed 1484 motifs and 36,737 protein domains. The tandem repeats were composed of 238,760 microsatellites (1–9 bp units) and a total of 6,658,938 bp in length, 106,286 minisatellites (10–99 bp units) and a total of 8,750,508 bp in length, and 10,745 satellites (≥ 100 bp units) with a total of 6,048,218 bp in length. Functional annotation of the predicted genes was performed against multiple databases, including NR, eggNOG, GO, KEGG, TrEMBL, KOG, SWISS‐PROT, and Pfam. Collectively, 97.7% of the genes were annotated to at least one of these databases (Table S4).

### 3.2 Genomic Divergence Between *N. macrophylla* and *N. cadamba*

**FIGURE 3:**
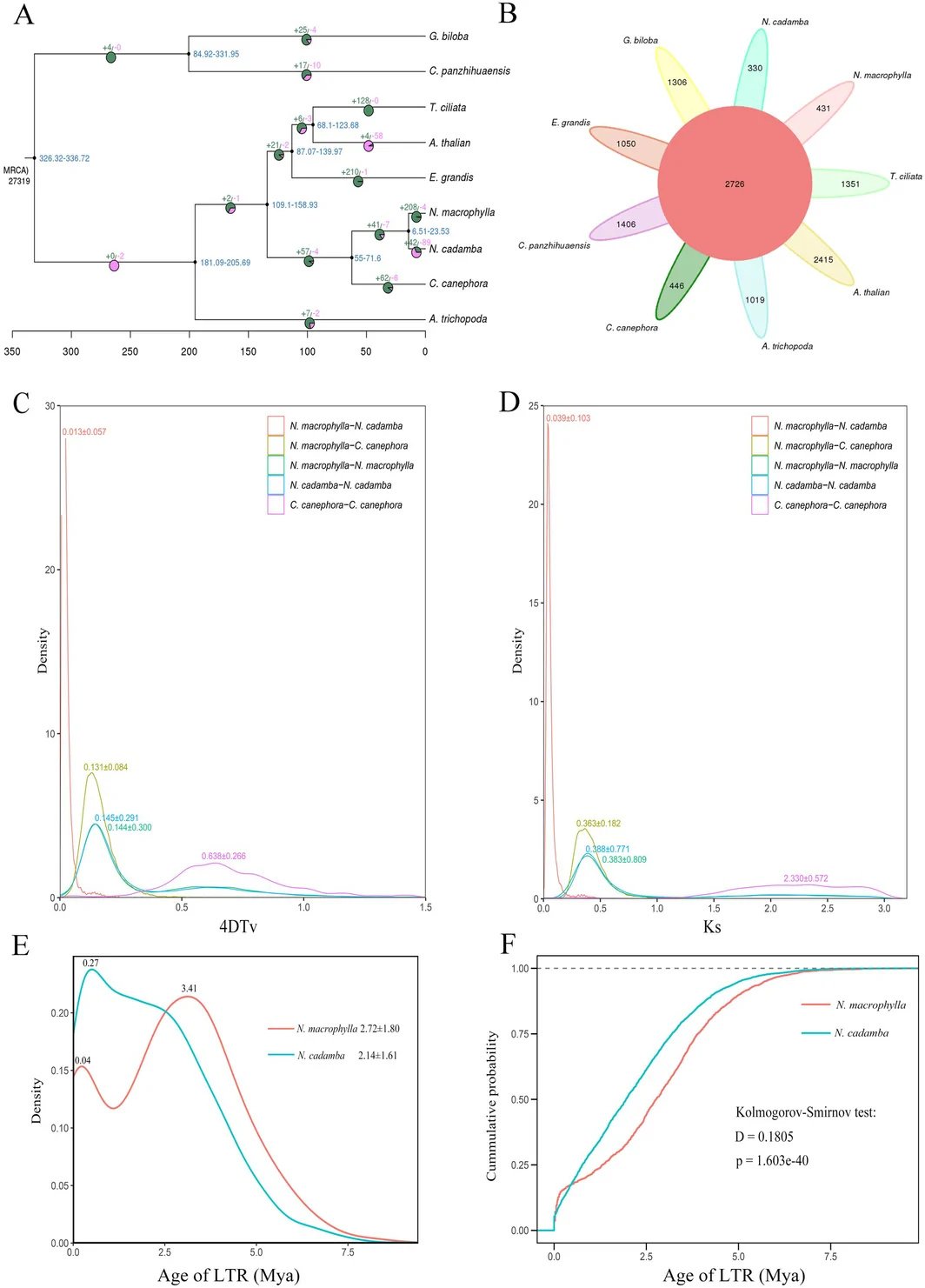
Genomic comparison between *N. macrophylla* and *N. cadamba* . (A) Phylogeny and comparison of gene families where the numbers of expanded gene families (with symbol “+”) are indicated in green and the number of contracted gene families (with symbol “−”) in red; In each pie chart, the proportion of expanded gene families is shown in blue, while the proportion of contracted gene families is shown in pink. (B) Clustering analysis of gene families among nine species; (C) Distribution of 4DTv estimates in *N. macrophylla* (11,200 estimates), *N. cadamba* (9712), and between them (19,830); (D) Distribution of *K*_s_ estimates in *N. macrophylla* (10,772), *N. cadamba* (9501), and between them (22,394); (E) Distribution of the LTR insertion times in *N. macrophylla* (2206) and *N. cadamba* (3955); (F) Kolmogorov–Smirnov test of LTR insertion times between the two species.

Analysis with ModelFinder, integrated within IQ‐TREE, identified the best‐fit substitution model, which was determined to be Jones‐Taylor‐Thornton + Empirical frequencies + Invariant sites + Gamma distribution 4 (JTT + F + I + G4). Phylogenetic analysis revealed that *N. macrophylla* and *N. cadamba* formed a clade among the nine species. The two *Neolamarckia* species diverged from their common ancestor approximately 6.51–23.53 Mya (Figure 3A). Their common ancestor diverged from *C. canephora* of the same family at approximately 55–71.6 Mya and further diverged from other fast‐growing tree species ( *T. ciliata* and *E. grandis* ). Collinearity analysis revealed that *N. macrophylla* and *N. cadamba* exhibited extensive collinear blocks, indicating their close phylogenetic relationship.

There were 3157 gene families identified in *N. macrophylla* , with 431 being species‐specific (Figure 3B). GO analysis revealed that these specific families are predominantly involved in biological processes, such as metabolic processes, biological regulation, and stress response. For the cellular components, they were primarily associated with subcellular structures including cellular parts, membranes, and organelles. For the molecular function, they were enriched in catalytic activity, binding functions, and transcription factor activity (Figure S2). Analysis of KEGG pathway indicated that they were significantly enriched in multiple pathways, including the biosynthesis of aminoacyl‐tRNA, valine, leucine, isoleucine, and lysine, and the metabolisms of butanoate, pyruvate, alanine, aspartate, glutamate, 2‐ococarboxylic acid, C5‐branched dibasic acid, and propanoate (Figure S3).

Compared with the common ancestor between *N. cadamba* and *N. macrophylla* , *N. macrophylla* expanded 208 gene families but contracted four gene families. GO analysis revealed that the expanded gene families were predominantly involved in biological processes related to metabolism, regulation, and stress response (Figure S4). For the cellular components, they were primarily localized to cell parts, membranes, and organelles. For molecular function, they were enriched in catalytic activity, binding functions, and transcription factor activity. KEGG pathway analysis demonstrated that the expanded gene families were prominently associated with monoterpenoid biosynthesis, indole alkaloid biosynthesis, galactose metabolism, and benzoxazinoid biosynthesis (Figure S5).

Estimates of 4DTv and *K*_s_ indicated that the evolutionary divergence between *N. cadamba* and *N. macrophylla* occurred more recently, with peak values of 0.013 for 4DTv and 0.039 for *K*_s_ (Figure 3C,D). One WGD event was detected in ancestral genomes of *N. macrophylla* and *N. cadamba* , with peak values of approximately 0.145 for 4DTv and 0.383 for *K*_s_. The peak value between *N. macrophylla* and *C. canephora* was approximately 0.131 for 4DTv and 0.363 for *K*_s_. One WGD event in *C. canephora* occurred much earlier, with peak values of approximately 0.638 for 4DTv and 2.330 for *K*_s_. These results indicated that the identified WGD event in ancestral genomes of the *Neolamarckia* species occurred close to their divergence from *C. canephora* .

*N. macrophylla* has substantially fewer repeat sequences in total than *N. cadamba* , especially the LTR retroelements that were lost after speciation (Table S16). The average insertion time was 2.72 ± 1.80 Mya for the retrotransposons with LTRs to integrate into the genome of *N. macrophylla* , and 2.14 ± 1.61 Mya into the genome of *N. cadamba* (Figure 3E). K‐S test indicated that the LTR insertion times were significantly earlier in *N. macrophylla* than in *N. cadamba* (*D*‐statistic = 0.1805, *p* value = 1.603 × 10^−40^) (Figure 3F).

**FIGURE 4:**
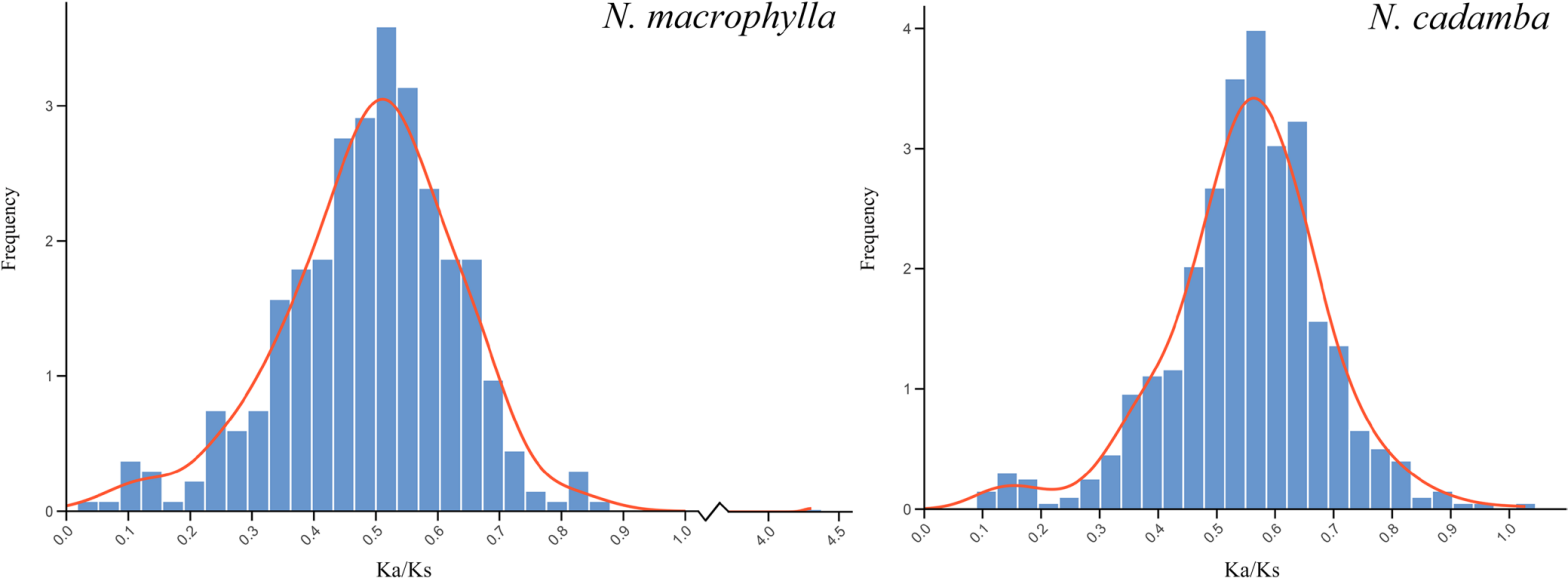
Histograms of the *K*_a_/*K*_s_ estimates for the 208 expanded gene families in *N. macrophylla* and 42 expanded gene families in *N. cadamba* .

To understand the evolutionary mechanisms underlying expanded genes, we estimated the *K*_a_/*K*_s_ ratios of 208 gene families of *N. macrophylla* and 42 of *N. cadamba* (Figure 3A). The average *K*_a_/*K*_s_ value was 0.501 ± 0.238 for *N. macrophylla* (after removing genes with *K*_s_ = 0 or *K*_a_ = 0) and 0.550 ± 0.135 for *N. cadamba* . Only one gene, *Nma22G009720.1*, was subject to positive selection (*K*_a_/*K*_s_ = 4.304) in the 208 expanded families of *N. macrophylla* (Figure 4). The function of this gene remains undetermined (Table S5). Five genes under strong purifying selection were annotated in function (Table S5): *Nma08G013120.1*, *Nma05G006810.1*, *Nma19G006250.1*, *Nma05G001020.1*, and *Nma09G003940.1*. Only one gene, *evm.model.Contig302*, was under weak positive selection (*K*_a_/*K*_s_ = 1.020) in 42 expanded gene families of *N. cadamba* and unknown in function (Table S5, Figure 4). Three genes under strong purifying selection were annotated (Table S5), including *evm.model.Contig271.116*, *evm.model.Contig12.291*, and *evm.model.Contig271.112*. These genes under positive selection and strong purifying selection were not directly involved in wood property traits.

### 3.3 Phenotypic and Genetic Variation of Wood Property Traits in *N. cadamba*

**FIGURE 5:**
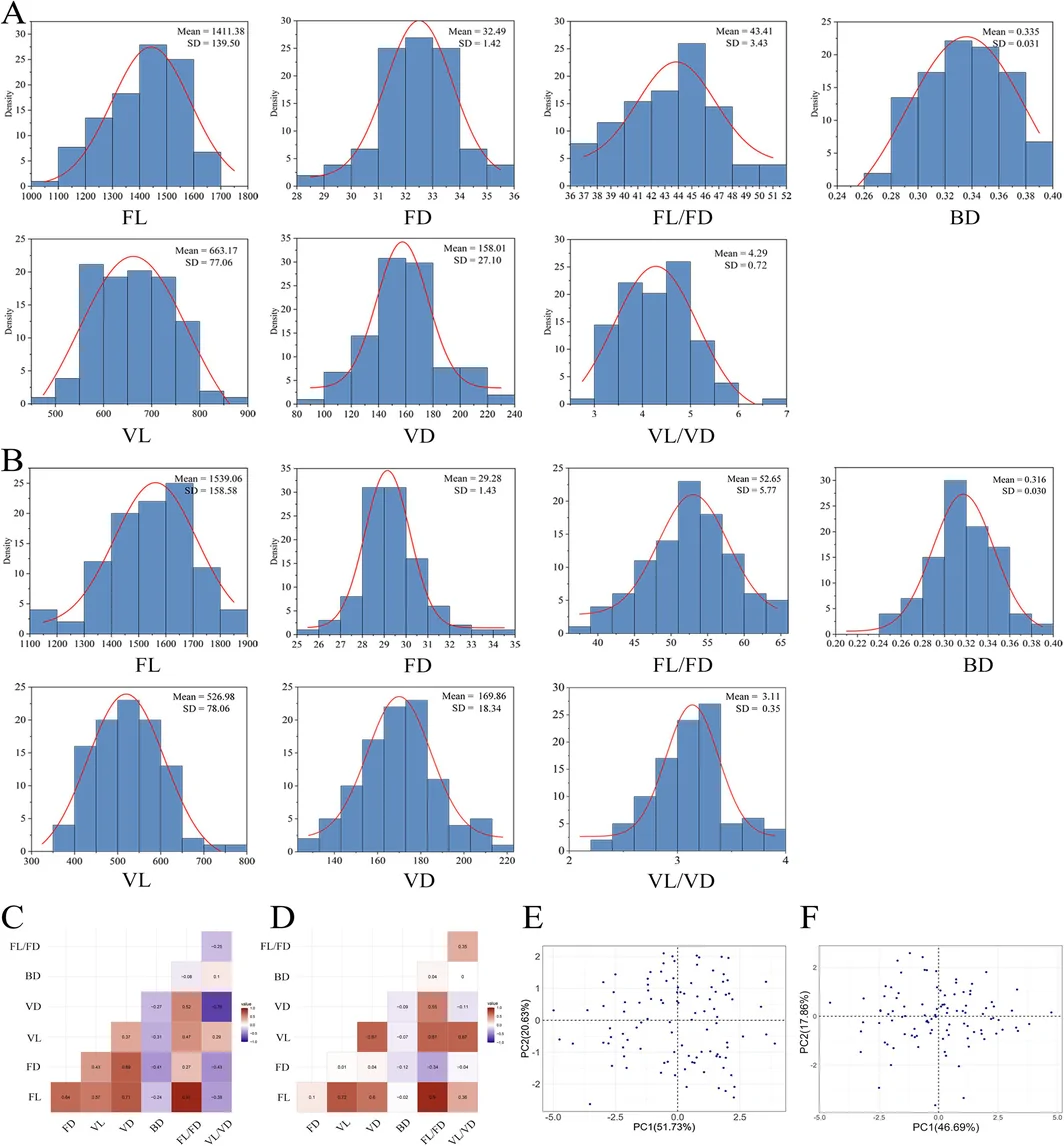
Phenotypic variations of wood property traits. (A) Histograms of wood property traits derived from samples at 7 years old; (B) Histograms of wood property traits derived from samples at 12 years old; (C) Heatmaps for correlations among wood property traits at 7 years old; (D) Heatmaps for correlations among wood property traits at 12 years old; (E) PCA with seven wood property traits at 7 years old; (F) PCA with seven wood property traits at 12 years old. BD, basic density; FD, fiber diameter; FL, fiber length; FL/FD, fiber length/fiber diameter ratio; PC1, the first principal component; PC2, the second principal component; VD, vessel diameter; VL, vessel length; VL/VD, vessel length/vessel diameter ratio.

We first briefly describe phenotypic variation of wood property traits of *N. cadamba* in two samples. The coefficient of variation (CV) across seven traits ranged from 4.38% to 17.15% at 7 years old and from 4.88% to 14.81% at 12 years old (Table S6). Almost all wood property traits exhibited normal distribution (*p* value > 5% from K‐S test) (Figure 5A,B, Table S6). In the first sample (age 7), FL had significantly positive correlations with FD, VL, VD, and FL/FD, but significantly negative correlations with BD and VL/VD. FD had significantly positive correlations with VL, VD, and FL/FD, but significantly negative correlations with BD and VL/VD. VL exhibited a significant positive correlation with FL/FD, VD, but significantly negative correlations with BD and VL/VD (Figure 5C, Table S7). In the second sample (age 12), FL was significantly positively correlated with VL, VD, FL/FD, and VL/VD. VL exhibited a significant positive correlation with VD and FL/FD. VD exhibited a significant positive correlation with FL/FD (Figure 5D, Table S8). PCA indicated that the wood property traits exhibited substantial phenotypic variation among individuals at ages 7 and 12, without subpopulation differentiation (Figure 5E,F).

**FIGURE 6:**
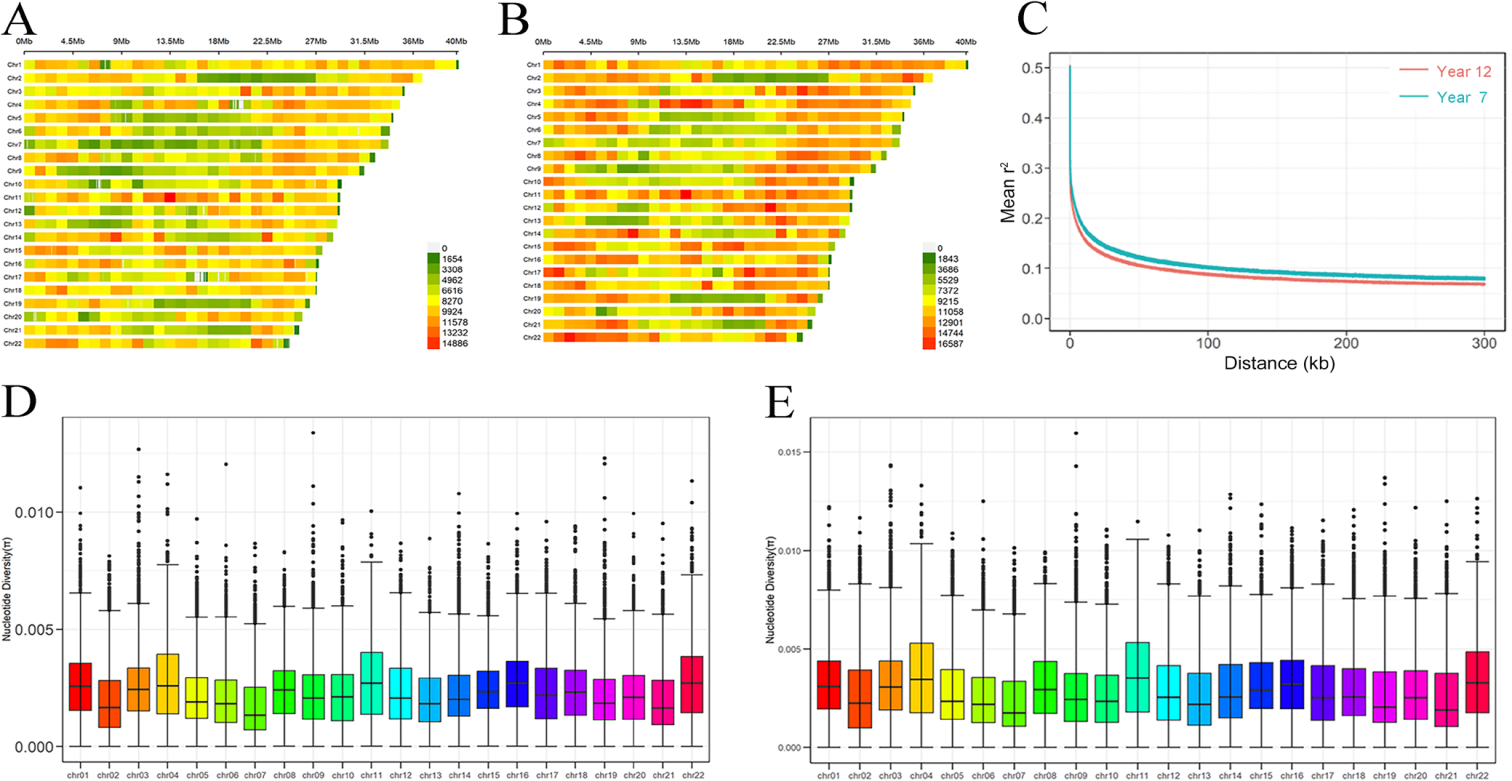
Genetic variation in the two samples of *N. cadamba* . (A) Genome‐wide SNP density visualized in 1‐Mb windows at age 7; (B) Genome‐wide SNP density visualized in 1‐Mb windows at age 12; (C) LD decaying pattern in two samples; (D) Box plots for nucleotide diversity at age 7; (E) Box plots for nucleotide diversity at age 12.

Genome resequencing showed substantial differences between the two samples in several assembly indices (Table 4). The samples at age 7 produced 14,052,220 SNPs and eventually obtained 3,835,469 SNPs after quality control mentioned in Section 2. The samples at age 12 produced 11,809,638 SNPs and eventually obtained 5,933,751 SNPs after quality control. This aligned with the expectation of different sampling schemes between the two samples. All SNPs exhibited 100% average coverage but were unevenly distributed across chromosomes (Figure 6A,B).

**TABLE 4:** A summary of genome resequencing in two samples of *N. cadamba* .

Item	The first sample (age 7)	The second sample (age 12)
Average raw data	11.85 ± 2.03 Gb	14.73 ± 3.40 Gb
Q20 (%)	≥ 94.31	≥ 95.95
Q30 (%)	≥ 86.34	≥ 89.82
GC (%)	35.78–38.19	35.66–38.00
Sequencing depth	9.35× to 24.3×	12.26× to 43.33×
Initial SNPs	14,052,220	11,809,638
SNPs after quality control	3,835,469	5,933,751

The average density of SNPs across 22 chromosomes was one SNP per 120.20 bp in the first sample (age 7), with the highest density on Chr11 (1 SNP per 96.15 bp) and the lowest on Chr09 (1 SNP per 133.78 bp). Chr21 contained the fewest markers (191,989 SNPs), whereas Chr01 possessed the most markers (373,020 SNPs) (Table S9). Similarly, the average density of SNPs across 22 chromosomes was one SNP per 93.81 bp in the second sample (age 12), with the highest density on Chr11 (1 SNP per 78.5 bp) and the lowest density on Chr09 (1 SNP per 108.0 bp). Chr21 contained the fewest markers (250,424 SNPs), whereas Chr01 possessed the most markers (470,311 SNPs) (Table S10).

Tests showed a total of 3,835,469 SNP loci under HWE in the first sample (age 7) and 5,933,751 SNP loci in the second sample (age 12). The two samples exhibited similar patterns across chromosomes (Figure S6). The SNPs under HWD (Hardy–Weinberg disequilibrium) accounted for 2.75% (Chr21) to 7.46% (Chr01) in the first sample and 3.29% (Chr21) to 7.55% (Chr04) in the second (Table S11).

Patterns of linkage disequilibrium (LD) decay were consistent with different sampling schemes between the two samples (Figure 6C). LD declined by half at approximately 16.02 kb in the first sample but approximately 24.43 kb in the second sample (age 12) (Figure 6C).

We estimated nucleotide diversity (*π*) within 1‐kb windows across chromosomes. The two samples showed similar patterns across chromosomes (Figure 6D,E). The average nucleotide diversity was 0.0023 ± 0.0015 in the first sample, ranging from 2.1879 × 10^−6^ to 0.0134, and 0.0030 ± 0.0020 in the second sample (age 12), ranging from 9.5487 × 10^−8^ to 0.0160. Both samples exhibited a skewed distribution of *π* values (Figure S7).

### 3.4 Identification and Evolution of Genes From GWAS on Wood Property Traits

**FIGURE 7:**
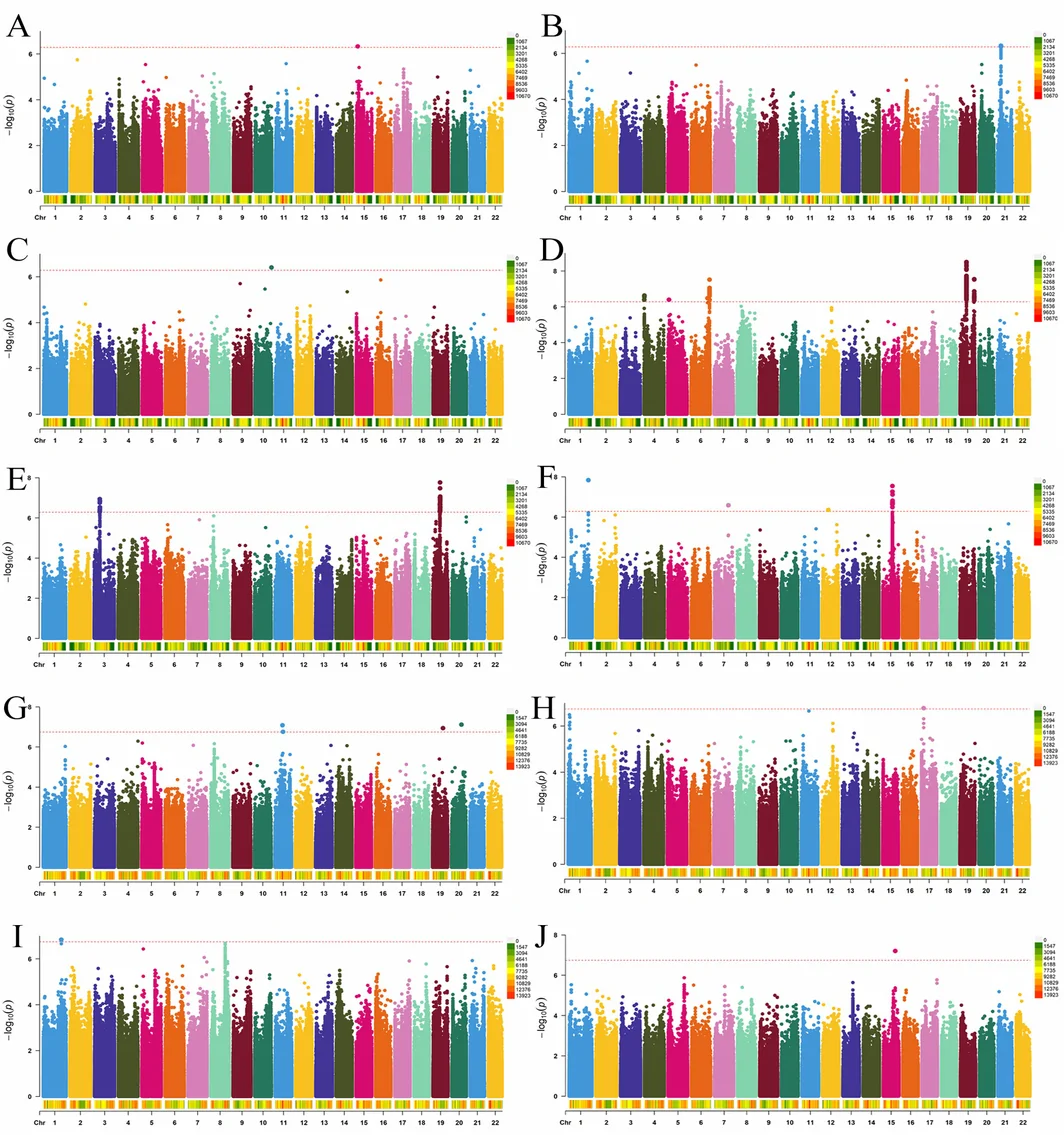
Manhattan plots for GWAS on wood traits. (A) FL; (B) FD; (C) VL; (D) VD; (E) BD; (F) VL/VD; (G) FD; (H) VD; (I) BD; (J) FL/FD. (A–E) Results from the first sample (age 7), whereas (G–J) refer to the results from the second sample (age 12). The red line in each figure indicates a significant threshold, 5.175 × 10^−7^ for the first sample and 1.791 × 10^−7^ for the second sample. The legends indicate the numbers of SNPs in different colors, and the bottom heatmap represents the SNP density distribution across chromosomes in each figure.

With the first sample (age 7), we identified 1 SNP significantly associated with FL, 3 SNPs with FD, 1 SNP with VL, 150 SNPs with VD, 59 SNPs with BD, and 16 SNPs with VL/VD (Figure 7A–F). With the second sample (age 12), we identified four SNPs significantly associated with FD, two SNPs with VD, one SNP with BD, and one SNP with FL/FD (Figure 7G–J). Specific positions of identified SNPs were provided in Table S12. Quantile‐quantile (Q‐Q) plots for each trait indicated that the observed *p* values were generally in agreement with predicted values under the null hypothesis, with deviations only occurring in the upper‐right tail, which collectively demonstrated the reliability of GWAS results (Figure S8).

We predicted candidate genes and annotated their functions within a 50‐kb region of upstream and downstream of each identified SNP. With the first sample (age 7), we identified 122 candidate genes that were closely linked to 230 SNPs, among which there were 4, 10, 2, 62, 19, and 25 genes related to FL, FD, VL, VD, BD, and VL/VD, respectively (Table S12). These genes were involved in diverse biological processes, including signal transduction and hormone regulation, epigenetic regulation and gene silencing, and RNA processing and regulation. They also participated in secondary metabolism and small‐molecule modifications, such as alkaloid and monoterpene biosynthesis, glycosylation, and posttranslational protein modifications. In addition, several candidate genes were closely associated with wood property formation. These genes were *NcSPS1*, involving carbon allocation; a glycosyltransferase gene, involving cell wall polysaccharide biosynthesis; and *NcDIR25*, participating in lignin biosynthesis (Table S13).

With the second sample (age 12), we annotated 32 candidate genes covering the 8 SNPs associated with four wood property traits (Table S12). There were 10 candidate genes closely related to FD, 4 key genes associated with VD, 8 candidate genes closely related to BD, and 10 candidate genes closely related to FL/FD. These genes were primarily involved in transcriptional regulation, RNA metabolism, and cell wall biosynthesis, collectively contributing to plant development, tissue differentiation, and structural integrity. For example, *NcCSLC5* encodes a xyloglucan glycosyltransferase 5 protein of the CSLC family that facilitates xyloglucan biosynthesis, a key polysaccharide in the plant cell wall (Table S13).

**FIGURE 8:**
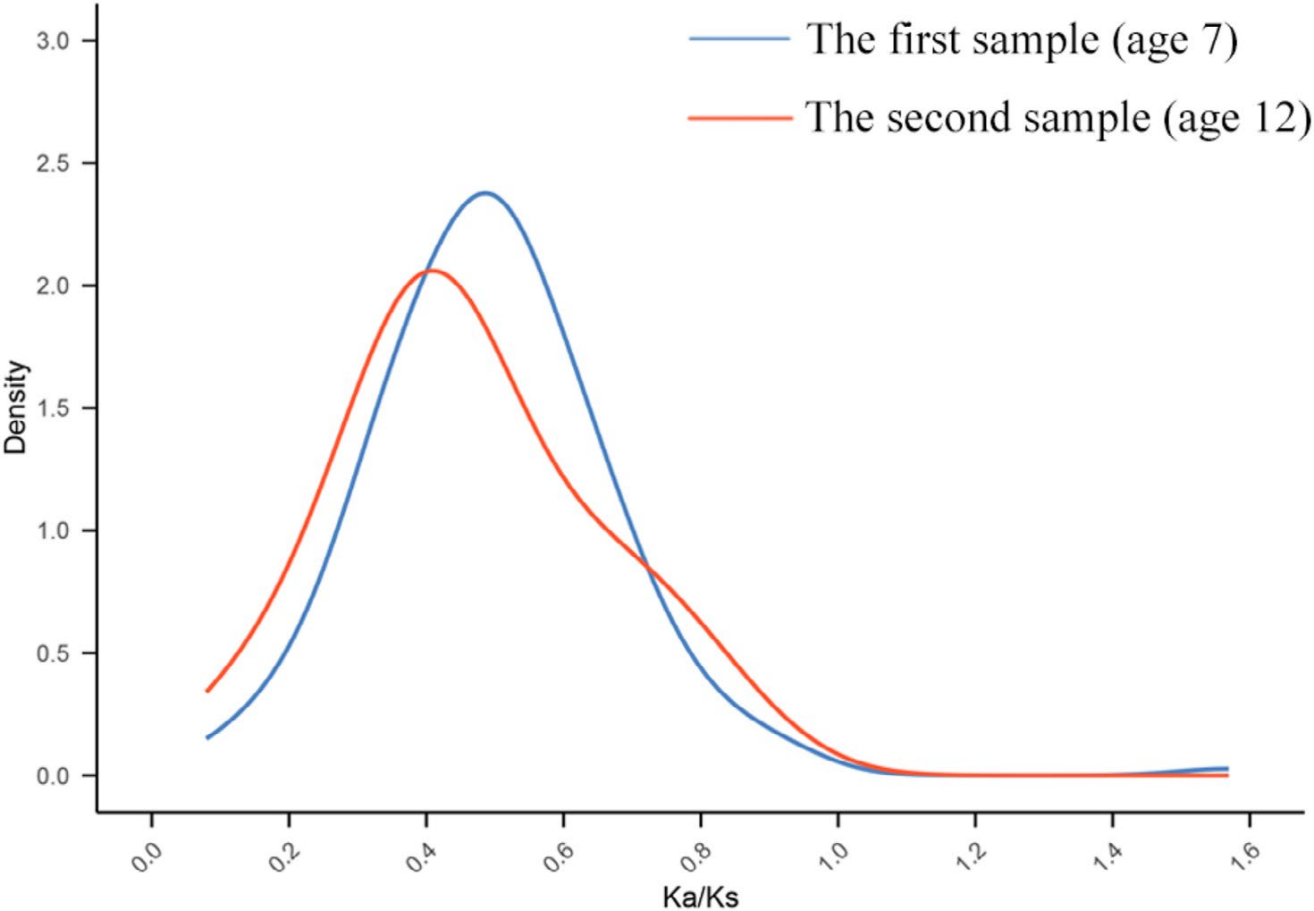
Distribution of *K*_a_/*K*_s_ estimates for genes associated with wood property traits. Two hundred homologous gene pairs in the first sample (age 7); 46 homologous gene pairs in the second sample (age 12).

Based on the genome sequences of *N. macrophylla* and *N. cadamba*, orthologous genes were obtained between the two species. We estimated the *K*_a_/*K*_s_ ratios of genes significantly associated with wood property traits (Figure 8). With the first sample, we obtained 200 estimates after removing those genes with *K*_s_ = 0. All genes except for 1 were under purifying selection, with the mean of 0.496 ± 0.176 and the range from 0.082 to 1.569. Generally, about 50.4% (= 1 − *K*_a_/*K*_s_) of nonsynonymous mutations were eliminated by natural selection. Two genes under strong selection (*K*_a_/*K*_s_ < 0.1) were *evm.model.Contig81.334.1* (*K*_a_/*K*_s_ = 0.091; APETALA2‐like protein 3) and *evm.model.Contig81.325.1* (*K*_a_/*K*_s_ = 0.082; annotated as uncharacterized protein). With the second sample, we obtained 46 estimates of *K*_a_/*K*_s_ ratios after removing genes with *K*_s_ = 0. All genes were under purifying selection, with the mean of 0.462 ± 0.185 and the range from 0.079 to 0.871. About 53.8% (= 1 − *K*_a_/*K*_s_) of nonsynonymous mutations were eliminated by natural selection.

### 3.5 Evolution of Genes Coding Key Enzymes for Lignin Biosynthesis

Here, we examined the evolutionary divergence between *N. cadamba* and *N. macrophylla* for the key genes controlling lignin biosynthesis. These genes are well mapped along pathways of different types of lignin biosynthesis. Our objective was to investigate natural selection on mutant alleles of these genes between the two species (Figure 3A). Based on the previous studies (Hu et al. 1999; Boerjan et al. 2003), the key enzymes include phenylalanine ammonia‐lyase (PAL), cinnamate 4‐hydroxylase (C4H), 4‐coumarate: CoA ligase (4CL), cinnamoyl‐CoA reductase (CCR), cinnamyl alcohol dehydrogenase (CAD), caffeic acid o‐methyltransferase (COMT), ferulate 5‐hydroxylase (F5H), coumarate sulfate esterase (CSE), p‐coumarate 3‐hydroxylase (C3H), and cinnamoyl‐coA o‐methyltransferase (CCoAOMT). Genes encoding these enzymes directly affect both lignin content and structural composition in plants (Boerjan et al. 2003).

**FIGURE 9:**
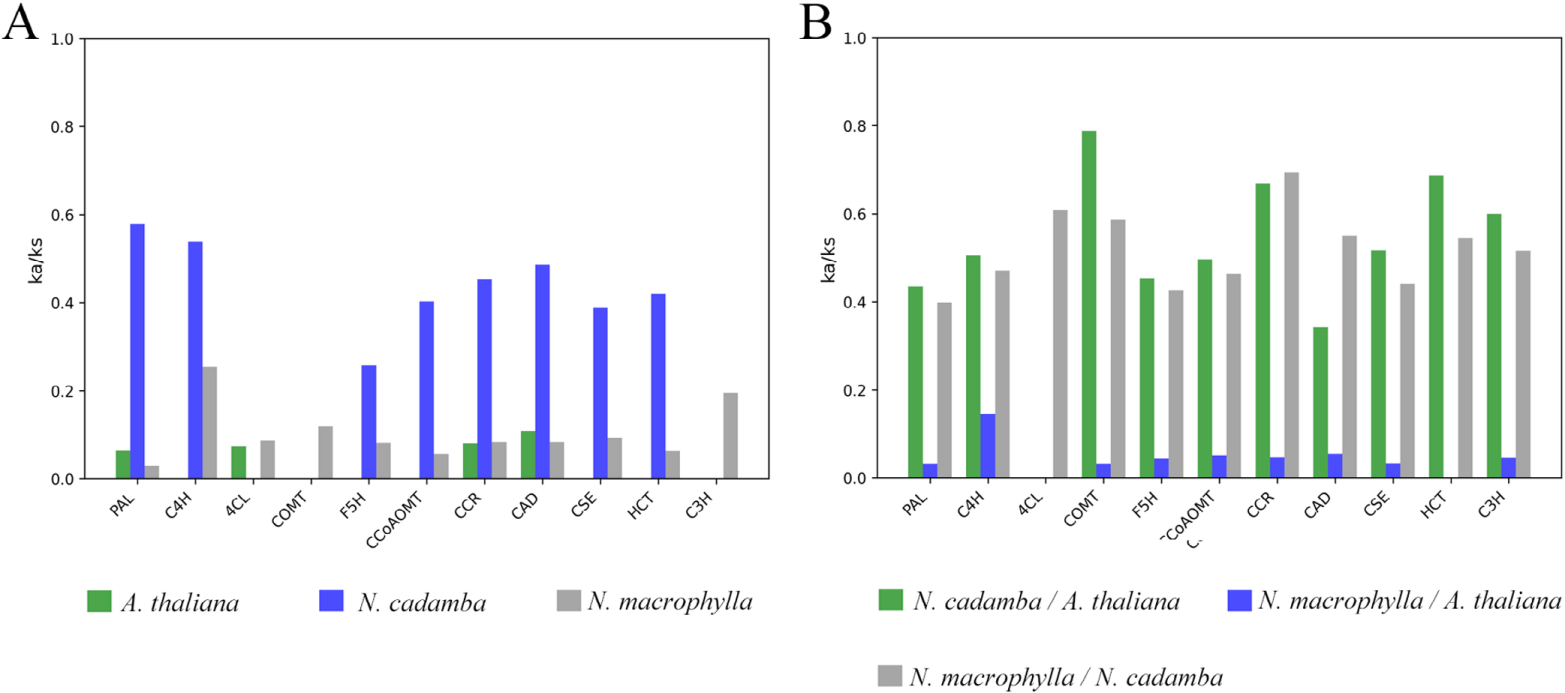
Natural selection pressure on genes for lignin biosynthesis. (A) *K*_a_/*K*_s_ ratios between gene family members (paralogous genes) within *A. thaliana* , *N. macrophylla* , and *N. cadamba* ; (B) *K*_a_/*K*_s_ ratios of orthologous genes among *A. thaliana* , *N. macrophylla* , and *N. cadamba* .

We identified 11 key lignin biosynthesis‐related gene families in both species, including *PAL*, *C4H*, *4CL*, *CCR*, *CAD*, *COMT*, *F5H*, *CSE*, *C3H*, and *CCoAOMT*. Compared with *A. thaliana* , species in the *Neolamarckia* genus were identified with more gene family members in almost all genes mentioned above (Table S14). We estimated *K*_a_/*K*_s_ values of paralogous gene family members within species and orthologous genes between species (Figure 9, Table S15). Results showed that intraspecific purifying pressure was generally strongest in *A. thaliana* , followed by *N. macrophylla* and *N. cadamba* . *N. macrophylla* was more conservative than *N. cadamba* in terms of precise and reliable lignin biosynthesis. Estimation of *K*_a_/*K*_s_ between species indicated that all genes for lignin biosynthesis were subject to purifying selection (Figure 9B, Table S15). *N. macrophylla* was more conservative than *N. cadamba* . Nevertheless, the relatively weak purifying selection existed in these genes between *N. macrophylla* and *N. cadamba* (*K*_a_/*K*_s_ = 0.398–0.693), which allowed maintenance of certain deleterious mutant genes in lignin biosynthesis.

**FIGURE 10:**
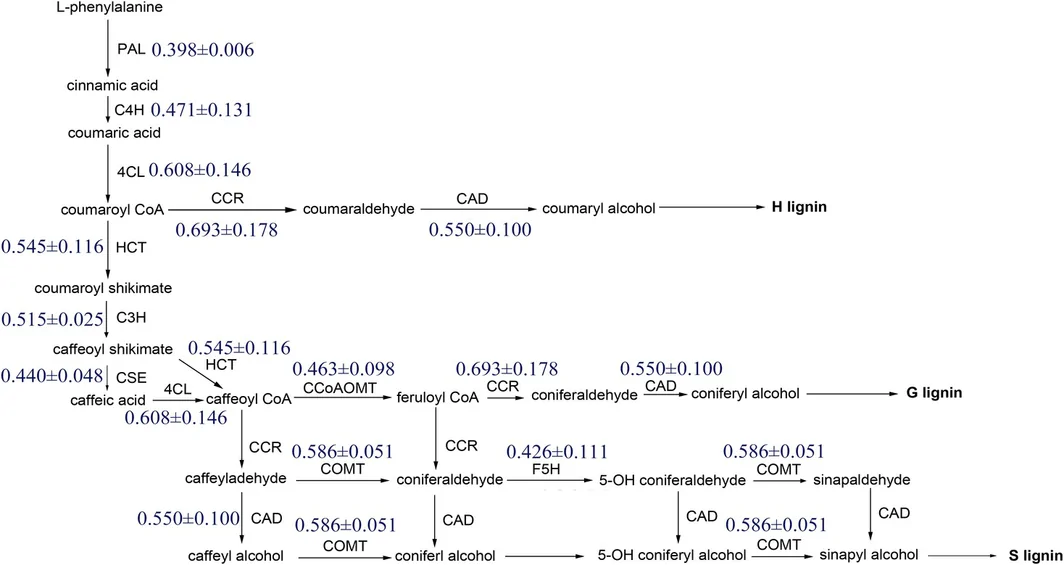
Natural selection pressure on genes encoding key enzymes in the pathway of lignin biosynthesis in genus *Neolamarckia*. Estimates of *K*_a_/*K*_s_ are indicated for genes encoding key enzymes. The pathway for hardwood lignin biosynthesis was derived from Bai et al. (2022).

When estimates of *K*_a_/*K*_s_ were mapped to the pathway of lignin biosynthesis (Figure 10), gene *PAL* had the strongest constraint from purifying selection and represented a crucial step for shifting to lignin biosynthesis in metabolism. Genes *C4H*, *CSE*, and *F5H* had comparable evolutionary rates between the two species under purifying selection, with more than 50% (1 − *K*_a_/*K*_s_) of deleterious mutations eliminated by natural selection. Enzymes coded by these genes occur in specific pathways. This was followed by genes *CAD*, *HCT*, *C3H*, and *COMT*, with < 50% of mutations eliminated by natural selection. Enzymes coded by these genes occur in multiple pathways for different types of lignin biosynthesis (p‐hydroxyphenyl, guaiacyl, and syringyl lignin). Similarly, genes *CCR* and *4CL* had relatively higher evolutionary rates, with < 40% of mutations eliminated by natural selection. Enzymes encoded by genes *CCR* and *4CL* are required in multiple intermediate steps of different types of lignin biosynthesis (Hu et al. 1999), and hence relatively loose constraints were imposed in nature.

## 4 Discussion

### 4.1 Genomic Divergence Between *N. cadamba* and *N. macrophylla*

In this study, we sequenced and assembled a high‐quality nuclear genome of *N. macrophylla* by integrating third‐generation HiFi with Hi‐C sequencing data, followed by Hi‐C scaffolding. The genome was approximately 598.43 Mb, anchored onto 22 chromosomes, and harbored 34,411 protein‐coding genes, 4843 noncoding RNAs, and 37 pseudogenes. This enriches the genomic resources of the *Neolamarckia* genus in the Rubiaceae family, the fourth largest family in flowering plants where only a few species have been sequenced, including *C. canephora* (Denoeud et al. 2014), *Gardenia jasminoides* (Xu et al. 2020), *Ophiorrhiza pumila* (Rai et al. 2021), and *N. cadamba* (Zhao et al. 2022). It also provides a valuable resource for conservation genomics and enables in‐depth comparative genomic analyses to elucidate evolutionary relationships among *N. macrophylla*, *N. cadamba* , and other Rubiaceae species.

Concerning the genomic divergences or similarities between *N. cadamba* and *N. macrophylla* and their biological significance, several specific conclusions can be drawn:

1. There are substantial differences in genomic characteristics between the two species. Based on k‐mer distribution analysis, the genome survey showed that *N. macrophylla* has a genome size of 556.38 Mb (vs. 754 Mb in *N. cadamba* ), a repeat content of 36.66% (vs. 54.29%) and a heterozygosity rate of 0.48% (vs. 0.69%). Specifically, this k‐mer‐based genome survey is fully independent of assembly quality and substantiates the genomic disparity between the two species. Furthermore, our genome assembly showed that *N. macrophylla* had a substantially smaller genome size (598.43 Mb vs. 744.5 Mb), fewer protein coding genes (34,411 vs. 35,461), a lower repetitive rate (36.66% vs. 54.29%), and a lower transposable element (TE) rate (40.8% vs. 52.9%) (Zhao et al. 2022).

*N. cadamba* acquired more and younger LTR retroelements in the genome than *N. macrophylla* (Figure 3F), contributing to the larger genome size. The reduced genomic complexity in *N. macrophylla* was likely attributable to its narrow natural distribution restricted to Sulawesi and Maluku island, where island isolation intensified genetic drift effects, potentially leading to the loss of repetitive sequences, particularly recent transposable element (TE) insertions, and fixation of deleterious mutations (Li et al. 2023). This can be implied from the distribution of *K*_s_ between LTR sequences within *N. macrophylla* (Figure 3E,F). This pattern is analogous to the genomic difference between *Toona ciliata* and *T. sinensis* (two fast‐growing and genetically related sympatric species) where *T. ciliata* has a smaller genome size and a more restrictive geographical distribution than *T. sinensis* (Wang, Xiao, He, et al. 2022). *T. sinensis* acquired more and younger LTR retroelements than *T. ciliata* . The LTR retroelement burst could enhance species adaptation to adverse environments, such as *Arabidopsis arenosa* to an elevational gradient (Wos et al. 2021), *Acer tsinglingense* to climate change (Meng et al. 2025), or birds to arid environments (Carotti et al. 2023). It should be noted that the aforementioned inference regarding the loss of repetitive sequences in relation to island isolation may be partially confounded by differences in genome assembly quality between the two species. Consequently, these conclusions could be preliminary and await more data collection.
2. The two species diverged at 6.51–23.53 Mya and coincided with the Miocene epoch, a period of Indonesian archipelago formation and the Mid‐Miocene Climatic Optimum, aligning with rapid island adaptive evolution. Using mitochondrial genomes, Xie et al. (2026) showed that the two species diverged at 6.757 Mya (2.472–12.107 Mya), supporting their recent divergence. One WGD event was detected prior to speciation, and the genome structure divergences were formed after speciation. Zhao et al. (2022) showed two WGD events occurred in *N. cadamba* : one recent WGD was the same as we detected, and the other was more distant. The distant WGD event in *N. cadamba* occurred at the time close to that of the WGD in *C. canephora* . Also, a comparison of 4DTv or *K*_s_ distribution implied that the WGD detected in our study occurred earlier than that of *T. ciliata* and *T. sinensis* (Wang, Xiao, He, Li, et al. 2022). The divergence times between *N. macrophylla* and *N. cadamba* were comparable to those between *T. ciliata* and *T. sinensis* (6–25 Mya; Wang, Xiao, He, Li, et al. 2022).
  A broader phylogeny indicated that the common ancestor of *N. cadamba* and *N. macrophylla* diverged from other species of wider geographical distributions in the Rubiaceae family at the time more than 55–71.6 Mya during the Paleocene‐Eocene epoch, characterized by Gondwanan breakup and tropical rainforest expansion. About 431 gene families specific to *N. macrophylla* suggested their potential roles for species adaptations to tropical islands, where nutrient leaching is common, possibly enhancing protein synthesis efficiency under limited nutrient availability.
3. Although many gene families (208) were expanded in *N. macrophylla* , all these genes except for a few genes were generally under purifying selection, implying the general convergent evolution between them at the genome scale. This was a similar situation to the expanded gene families (42) in *N. cadamba* that were under purifying selection. Nevertheless, the strengths of purifying selection were moderate for most expanded gene families in either species, as indicated by the *K*_a_/*K*_s_ estimates (Figure 4), implying that certain levels of deleterious mutations were maintained. Functional annotations showed that most expanded gene families were related to metabolism, regulation, and stress response (Figures S4 and S5). The deleterious mutant genes of *N. macrophylla* could reduce population adaptation to habitats and species geographic distribution. Those gene families under strong purifying selection primarily participate in the synthesis of secondary metabolites and defense compounds, suggesting they perform critical functions within specific secondary metabolic networks. Only a few gene families exhibiting positive selection could drive species adaptive divergence or enhance novel functions, but their relations to wood property traits remain to be determined. The general conclusion is that most expanded gene families in *N. macrophylla* potentially confine its adaptation to more restrictive niches, analogous to the situation of *T. ciliata* , which has more expanded gene families but is restricted to a smaller distribution range than *T. sinensis* (Wang, Xiao, He, Li, et al. 2022).

### 4.2 Evolution and Implications of Genes Associated With Wood Property Traits

The similarity between *N. cadamba* and *N. macrophylla* in basic wood property traits can also be inferred from previous phenotypic comparisons (Cahyono et al. 2016; Anna et al. 2020; Que et al. 2022). The present study explored evolutionary mechanisms to explain this similarity. We identified 122 and 32 candidate genes with the first (age 7) and the second (age 12) samples, respectively. The candidate genes derived from the first sample played roles in diverse biological processes, including wood property formation. The candidate genes derived from the second sample covered eight significant SNPs associated with four wood property traits. These genes participated in transcriptional regulation, RNA metabolism, and cell wall biosynthesis, and directly or indirectly affected wood property traits (Table S13).

One striking feature is that almost all candidate genes were under strong or moderate purifying selection. This effectively impeded evolutionary divergence in genes associated with wood property traits between *N. cadamba* and *N. macrophylla* and enhanced convergent evolution of these genes. Gene *evm.model.Contig81.334.1* (APETALA2‐like protein 3) was under exceptionally strong purifying selection (*K*_a_/*K*_s_ < 0.1) and was an important regulatory factor in plant growth and development, involving multiple biological processes, such as flowering time, meristem development, and organ formation. AP2‐like proteins participate in the regulation of shoot apical meristem (SAM) and floral meristem development. By modulating cell proliferation, differentiation, and fate determination within meristems, they influence meristem size, shape, and function (Zhou et al. 2021; Bertran Garcia de Olalla et al. 2024).

The intraspecific purifying selection was the strongest in *A. thaliana* , followed by *N. macrophylla* and *N. cadamba* . This likely reflects fundamental differences in life‐history strategy and genomic architecture between herbaceous and woody plants (Figure 9). As an annual herb, *A. thaliana* relies on a relatively small, nonredundant core set of lignin biosynthesis genes that are essential for structural support in stress responses. The lack of extensive gene duplication means that deleterious mutations in these genes have immediate fitness consequences, resulting in strong purifying selection. In contrast, woody species have experienced lineage‐specific expansions of lignin‐related gene families through whole‐genome and tandem duplication, as demonstrated in comparative genomic analyses of woody lineages (Su et al. 2025). Increased gene copy number introduces functional redundancy, allowing sub‐functionalization or specialization among duplicates and thereby relaxing purifying selection on individual gene copies. This evolutionary framework provides a mechanistic explanation for the weaker average purifying selection observed in *Neolamarckia* species. The modest difference between *N. macrophylla* and *N. cadamba* may further reflect fine‐scale ecological adaptation, consistent with species‐specific life histories and habitat distributions. Together, these results suggest that variation in intraspecific selection pressure on lignin genes is shaped by the interplay between life form, gene family evolution, and functional redundancy.

Similar results were detected on the 11 gene families encoding key enzymes in the lignin biosynthesis pathway. All genes were under moderate strengths of purifying selection (*K*_a_/*K*_s_ = 0.398–0.693), implying constraints on lignin synthesis genes in both species. The two species tend to show convergent evolution in these genes. This aligns with the basic function of lignin in plant biology (Boerjan et al. 2003). The lignin biosynthesis pathway involves a precisely coordinated enzyme cascade whose high evolutionary conservation (manifested by strong purifying selection) represents an evolutionary imperative, ensuring functional stability and reliability in this essential process. Purifying selection on these key genes could alter lignin content between the two species. The gene‐level gradient in selection pressure may affect this phenotypic variation, although experimental evidence has not yet been recorded in the genus *Neolamarckia*. A few reports showed phenotypic variation between the two species (Wu et al. 2026), with 28.57% (Latib and Kasim 2014; Rahman et al. 2021) or 24.92% ± 0.12% (Amirta et al. 2016) in *N. cadamba* , and 24.04% ± 0.17% in *N. macrophylla* (Mukhdlor et al. 2021). A recent study showed that *N. macrophylla* had a higher Klason lignin content (25.68%–26.18%) than *N. cadamba* (22.80%–23.89%) (Arisandi et al. 2025). We speculate that the restricted island distribution of *N. macrophylla* may impose distinct ecological pressures that favor tighter functional constraint on lignin components, potentially contributing to different lignin depositions. However, further functional and ecological studies are required to test this hypothesis.

The pattern of selection strengths, *PAL* > *C4H/CSE* > *CCR/4CL*, likely reflects different degrees of functional importance and evolutionary conservation among these genes. *PAL*, acting as the initial and rate‐limiting enzyme, controls the entry flux into the pathway of lignin biosynthesis and is crucial for cell wall formation and mechanical strength. Its sequence is highly conserved and typically under the strongest purifying selection. *C4H* and *CSE*, catalyzing subsequent steps and participating in metabolic crosstalk, experience slightly relaxed selective constraints. In contrast, downstream genes like *CCR* and *4CL*, primarily responsible for providing precursors for lignin monomers, may have more specialized or tissue‐specific roles and are thus subject to relatively weaker purifying selection.

tRNAs and rRNAs primarily function in protein translation, whereas miRNAs are recognized as key posttranscriptional regulators in secondary growth and wood development. A comparison of noncoding RNA revealed that *N. cadamba* has fewer rRNA (3284) and tRNA (666) but more miRNA (1053), snRNA (2701), and snoRNA (2560) than *N. macrophylla* (Zhao et al. 2022). Such differences could likely cause divergence in wood development between the two species. Among the identified miRNAs, miR166, miR390, and miR156 were reported in other plants to target genes involved in cellulose or lignin biosynthesis, suggesting their potential roles in regulating wood formation. Previous studies showed that the expression of lignin biosynthesis pathway genes can be regulated by alternative splicing, microRNA, and long noncoding RNA, as evidenced in *Populus* (Quan et al. 2019; Zhang et al. 2020). A few transcription factors, such as NAC, MYB, and WRKY TFs, regulate the expression of *PAL* and *CCoAOMT* genes in *Populus* (Zhang et al. 2020). In *Populus*, miR156 indirectly repressed lignin biosynthesis by inhibiting the expression of *SPL*, resulting in reduced activity of downstream lignin biosynthetic genes and decreased lignin accumulation (Wang et al. 2020). PagmiR166c targets the auxin biosynthesis‐related gene *PagECH2* and modulates cambial differentiation toward secondary xylem, cell expansion, lignin deposition, and secondary cell wall formation through distinct auxin signaling pathways (Zhao et al. 2025). In addition, overexpression of miR390b significantly increases the number of fiber cells along the longitudinal axis, and the miR390–TAS3–ARFs pathway plays a crucial role in coordinating stem elongation and vascular development in *Populus* (Shi et al. 2022). Thus, further study on transcriptional and posttranscriptional mechanisms could provide insights into differences in wood property traits between the two species.

It is important to emphasize that the candidate genes identified by GWAS provided valuable resources for genetic improvement of *N. cadamba* . These 154 identified candidate genes were associated with different wood property traits. They were primarily involved in signal transduction, hormone regulation, epigenetic regulation, RNA processing, secondary metabolism, and small‐molecule modifications. Notably, several genes were closely connected to wood formation, including *NcSPS*1, a glycosyltransferase gene, and *NcDIR*25 (lignin relative). These would provide a molecular basis for marker‐assisted breeding and targeting key wood property traits. Additionally, the functional annotation of candidate genes provided mechanistic insights into trait regulation, which could be used to guide genetic improvement via gene editing.

A few cautions deserve attention regarding GWAS. One is the small sample sizes that allow for screening for a limited number of SNPs associated with wood property traits. SNPs with small effects on wood property traits were difficult to detect due to sample size effects. Since the samples were taken from a provenance progeny test under equal age and a relatively uniform environment, the effects of environment and genotype by environment were effectively removed, improving the power of screening for reliable SNPs of certain effects. The second caution is the impacts of kinship on SNP identifications. We minimized this impact through strict SNP quality control and inclusion of kinship in GWAS. The third caution is the difference between the two samples due to different sampling schemes. Both samples displayed genetic variation among individuals and did not have stratification, as indicated by PCA. However, distinct sampling schemes could bring about different results of GWAS. For the first sample (age 7), trees with large differences in size were collected within each half‐sib. For the second sample (age 12), trees were randomly selected within each half‐sib family. Besides the potential age effects, the different sampling schemes could affect GWAS. A larger sample of resequencing in future study could enhance detection of more SNPs associated with wood property traits.

## 5 Conclusions

This study presented the first high‐quality chromosome‐level assembly of the *N. macrophylla* genome, spanning 598.43 Mb with contig N50 and scaffold N50 lengths of 23.41 and 24.54 Mb, respectively. *N. macrophylla* had a smaller genome size and fewer predicted protein‐coding genes (34,411) than *N. cadamba* . Comparative genomic analysis revealed that one WGD event occurred prior to the split of the two species (6.51–23.53 Mya). *N. macrophylla* had substantially expanded more gene families than *N. cadamba* , and most gene families were under purifying selection. A total of 204 *N. cadamba* accessions collected from different provenances were resequenced for GWAS on wood property traits. All identified candidate genes except for a few genes were under purifying selection, effectively impeding evolutionary divergence between the two species in wood property traits. Further analysis demonstrated that the gene families encoding 11 enzymes (*PAL, C4H, 4CL, CCR, CAD, COMT, F5H, CSE, C3H, CCoAOMT*) along the lignin biosynthesis pathway were under purifying selection, indicating strong evolutionary conservation of these key genes. Gene families for lignin biosynthesis tended toward convergent evolution, underscoring the fundamental importance of the lignin synthesis pathway throughout their evolutionary history.

## Funding

This work was jointly supported by the 14th Five‐Year National Key Research and Development Program of China (Grant No. 2022YFD2200205), administered by the China Rural Technology Development Center, Ministry of Agriculture and Rural Affairs of the People's Republic of China, and by South China Agricultural University (Grant No. 4400‐K16013).

## Conflicts of Interest

The authors declare no conflicts of interest.

## Supporting information


**Figure S1:** K‐mer frequency distribution in the *Neolamarckia macrophylla* genome. The horizontal axis represents the depth of the K‐mer, and the vertical axis represents the frequency of the K‐mer at a given depth.
**Figure S2:** GO enrichment analysis of species‐specific gene families in *Neolamarckia macrophylla*.
**Figure S3:** KEGG analysis of species‐specific gene families in *Neolamarckia macrophylla*.
**Figure S4:** GO enrichment analysis of expanded gene families in *Neolamarckia macrophylla*.
**Figure S5:** KEGG analysis of expanded gene families in *Neolamarckia macrophylla*.
**Figure S6:** Distribution of *p*‐values from Hardy–Weinberg equilibrium (HWE) testing in the two samples of *Neolamarckia cadamba*.
**Figure S7:** Distributions of nucleotide diversity (*π*) per 1‐kb window size in two samples of *Neolamarckia cadamba*.
**Figure S8:** Quantile‐quantile plots for GWAS on wood traits of *Neolamarckia cadamba* . (A) FL; (B) FD; (C) VL; (D) VD; (E) BD; (F) VL/VD; (G) FD; (H) VD (I) BD; (J) FL/FD. (A–F) GWAS in the first sample, whereas (G–J) refer to the results in the second sample.
**Table S1:** The sequencing data of HiFi in *Neolamarckia macrophylla*.
**Table S2:** The sequencing data of Hi‐C in *Neolamarckia macrophylla*.
**Table S3:** The genome assembly data from Hi‐C analysis in *Neolamarckia macrophylla*.
**Table S4:** Gene annotation based on different databases from sequencing of *Neolamarckia macrophylla* genome.
**Table S5:** Annotations of genes under strong purifying and positive selection in expanded gene families.
**Table S6:** Phenotypic variation of wood traits in two samples of *Neolamarckia cadamba* for resequencing.
**Table S7:** Correlations among wood property traits for the first sample (age 7) of *Neolamarckia cadamba*.
**Table S8:** Correlations among wood property traits for the second sample (age 12) of *Neolamarckia cadamba*.
**Table S9:** Distribution of SNPs across chromosomes in the first sample (age 7) of *Neolamarckia cadamba*.
**Table S10:** Distribution of SNPs across chromosomes in the second sample (age 12) of *Neolamarckia cadamba*.
**Table S11:** Distribution of SNPs under Hardy–Weinberg disequilibrium (HWD) across chromosomes in two samples of *Neolamarckia cadamba*.
**Table S12:** Chromosomal positions of SNPs associated with wood property traits from GWAS of *Neolamarckia cadamba*.
**Table S13:** Annotations of candidate genes covering the SNPs associated with wood traits within 50 kb in two samples of *Neolamarckia cadamba*.
**Table S14:** Comparison of gene‐family members relevant to lignin biosynthesis among *Arabidopsis thaliana* , *Neolamarckia cadamba* , and *Neolamarckia macrophylla*.
**Table S15:** Detection of selective pressure on gene families for lignin biosynthesis in *Neolamarckia*.
**Table S16:** Comparison of repeat contents in *Neolamarckia macrophylla* versus *Neolamarckia cadamba* genome.

## Data Availability

The genome sequences of *N. macrophylla* are available under accession no. CNP0007944 in the CNGB Nucleotide Sequence Archive (CNSA: https://db.cngb.org/data_resources/?query=CNP0007944), including raw sequence reads, genome assembly files, and ncRNA files. The resequencing data of 100 individuals in the second sample of *N. cadamba* have been archived in the CNGB Nucleotide Sequence Archive under accession no. CNP0007971 (CNSA: https://db.cngb.org/data_resources/?query=CNP0007971).
