# Supplementary material for: Identification and Characterization of Genes Associated With Wood Property Traits via Genome Sequencing in Fast‐Growing Species of Genus Neolamarckia
Source: Evol Appl. 2026 Aug 24;19(8):e70314. doi: 10.1111/eva.70314 (PMC13501114; doi:10.1111/eva.70314)
Supplement: Supplementary file 1 [file EVA-19-e70314-s001.docx]

**Supporting information**

**Identification and characterization of genes associated with wood property traits via genome sequencing in fast-growing species of genus *Neolamarckia***

**Materials and Methods:**

**I. Genome sequencing of *N. macrophylla*;**

**II. RNA sequencing of *N. macrophylla*;**

**III. Genome resequencing of *N. cadamba* for GWAS.**

**Table S1-S16**

**Figure S1-S8**

**Materials and Methods**

**I. Genome sequencing of *N. macrophylla***

(1) Genome Survey Sequencing Library Construction

The genome survey library construction was performed according to the standard protocol provided by Illumina. First, the genomic DNA was fragmented to the target size (350 bp) using mechanical fragmentation (ultrasonication). Subsequently, a small fragment sequencing library was constructed through steps including end repair, A-tailing, adaptor ligation, size selection of target fragments, and PCR amplification. The library fragment size and concentration were determined using the Qseq400 Bio-Fragment Analyzer and Qubit fluorometer, respectively. Following successful quality control, the library was immobilized onto a flow cell, where bridge amplification was performed to cluster the library templates. Paired-end sequencing (150 bp reads, PE 150) was then conducted on an Illumina NovaSeq 6000 platform. Raw Illumina short-read sequences underwent quality filtering using fastp v0.23.4 (Chen et al. 2018) with parameters: -q 10 -u 50 -y -g -Y 10 -e 20 -l 100 -b 150 -B 150. The quality-filtered reads were subsequently employed for genome size estimation.

(2) Hi-C and High-fidelity (HiFi) Library construction and sequencing

For Hi-C sequencing, quality-certified DNA was processed via in situ Hi-C methodology: cells were crosslinked with formaldehyde (1%) to preserve 3D chromatin architecture through protein-DNA/DNA-DNA interactions, digested with HindIII restriction endonuclease to generate cohesive ends, repaired with biotinylated nucleotide incorporation, circularized through intramolecular ligation to capture interacting fragments, decrosslinked, purified, sheared to 300–700 bp fragments, and enriched using streptavidin-coated magnetic beads for library construction. After library construction was completed, the concentration and insert size of the library were determined using a Qubit 2.0 fluorometer and an Agilent 2100 Bioanalyzer, respectively. The effective concentration of the library was accurately quantified by quantitative real‑time PCR (qPCR) to ensure library quality. Quality-passed libraries were sequenced on an Illumina X-PIUS platform employing Sequencing By Synthesis (SBS) with 150-bp paired-end reads (PE150).

High-fidelity (HiFi) sequencing libraries were prepared according to the manufacturer's standard protocol for Circular Consensus Sequencing (CCS). Genomic DNA was sheared using g-TUBE devices (Covaris), followed by DNA damage repair. End repair was performed, after which SMRTbell hairpin adapters were ligated. Subsequent exonuclease digestion removed unligated fragments. Size selection of target fragments was conducted using the Blue Pippin system (Sage Science) to generate final sequencing libraries. Libraries passing quality control metrics were subjected to sequencing on the PacBio Revio platform.

**II. RNA sequencing of *N. macrophylla***

Total RNA was subjected to poly(A) selection using oligo(dT)-conjugated magnetic beads to enrich eukaryotic mRNA. Enriched mRNA was fragmented in Fragmentation Buffer, followed by first-strand cDNA synthesis primed with random hexamers. Second-strand cDNA synthesis was then performed using buffer, dNTPs, RNase H, and DNA polymerase I, with subsequent purification using AMPure XP beads. The purified double-stranded cDNA underwent end repair, A-tailing, and adapter ligation, followed by size selection with AMPure XP beads. Final library amplification was achieved through PCR enrichment. Constructed libraries were evaluated for concentration via Qubit 2.0 Fluorometer and insert size distribution using the Agilent 2100 Bioanalyzer. Effective library concentration was precisely quantified through qPCR to ensure quality control. Libraries passing quality thresholds were sequenced on an Illumina platform with 150-bp paired-end reads (PE150).

**III. Genome resequencing of *N. cadamba* for GWAS**

The experimental procedure followed the standard protocol provided by Illumina company, including DNA sample quality check, library construction, library quality testing, and library sequencing. Genomic DNA was extracted using the cetyltrimethylammonium bromide (CTAB) method (Doyle and Doyle 1987). The concentration and purity of gDNA were determined using a Nanodrop 2000 spectrophotometer and a Qubit 3.0 fluorometer (Thermo Fisher Scientific, Massachusetts USA). DNA integrity was evaluated by 1.0% agarose gel. The qualified DNA samples had a concentration of more than 5 ng/ul and the total amount was not less than 0.5 μg, with the main diffusion bands of more than 6 kb in size for integrity and no obvious impurities.

After sample quality control (QC), a total amount of 0.2 μg DNA per sample was used as input material for the DNA library preparations. Briefly, genomic DNA sample was fragmented by sonication to a size of 350 bp. Then DNA fragments were endpolished, A-tailed, and ligated with the full-length adapter for Illumina sequencing, followed by further size selection and PCR amplification. PCR products were purified by AMPure XP system (Beverly USA). Subsequently, library quality was assessed on the Agilent 5400 system (Agilent, USA) and quantified by QPCR (1.5 nM).

The qualified libraries were pooled and sequenced on Illumina platforms with PE150 strategy, according to effective library concentration and data amount required.

The raw data from original fluorescence image files obtained from Illumina platform are transformed and recorded in FASTQ (Cock et al. 2010) format. Sequence artifacts, including reads containing adapter contamination, low quality nucleotides and unrecognizable nucleotides (N), were controlled by following data processing:

(1) Discard the paired reads if either one read contains adapter contamination (>10 nucleotides aligned to the adapter, allowing ≤ 10% mismatches);

(2) Discard the paired reads if more than 10% of bases are uncertain in either one read;

(3) Discard the paired reads if the proportion of low quality (Phred quality < 5) bases is over 50% in either one read.

Reads were mapped to the reference genome by Burrows Wheeler Aligner (BWA) (Li et al. 2009a) software to get the original mapping results stored in BAM format (parameter: mem -t 4 -k 32 -M). Then, the results were deduplicated by SAMtools (Li et al. 2009b) (parameter: rmdup) and Picard (http://broadinstitute.github.io/picard/).

The raw SNP/InDel sets were called by SAMtools with the parameters as ‘-C 50 - mpileup -m2 -F 0.002 -d 1000’. Then we filtered these sets using the following criteria: (1) The depth of the variate position >4 and (2) The mapping quality >20.

CNVs were detected by CNVnator (Abyzov et al. 2011) to acquire potential deletion and duplication (parameter: - call 100). SVs were detected by BreakDancer(Chen et al. 2009). ANNOVAR (Wang et al. 2010) was used for functional annotation of variants. The UCSC known genes were used for gene and region annotations.

**References**:

Abyzov, A., et al., 2011. CNVnator: an approach to discover, genotype, and characterize typical and atypical CNVs from family and population genome sequencing. Genome Res 21(6):974-984. https://doi.org/10.1101/gr.114876.110

Chen, K., et al., 2009. BreakDancer: an algorithm for high resolution mapping of genomic structural variation. Nat Methods 6(9):677-681. https://doi.org/10.1038/nmeth.1363

Chen S, et al., 2018. fastp: an ultra-fast all-in-one FASTQ preprocessor. Bioinformatics 34(17):i884–i890. https://doi.org/10.1093/bioinformatics/bty560

Cock, P.J., et al., 2010. The Sanger FASTQ file format for sequences with quality scores, and the Solexa/Illumina FASTQ variants. Nucleic Acids Res 38(6):1767-1771. https://doi.org/10.1093/nar/gkp1137

Doyle, J.J., Doyle, J.L., 1987. A rapid DNA isolation procedure for small quantities of fresh leaf tissues. Phytochem Bull 19:11–15.

Li, H., Durbin, R., 2009. Fast and accurate short read alignment with Burrows-Wheeler transform. Bioinformatics 25:1754-1760. https://doi.org/10.1093/bioinformatics/btp324

Li, H., et al., 2009. The Sequence Alignment/Map format and SAMtools. Bioinformatics 25(16):2078-9. https://doi.org/10.1093/bioinformatics/btp352

Wang, K., et al., 2010. ANNOVAR: functional annotation of genetic variants from high-throughput sequencing data. Nucleic Acids Res 38(16):e164.

https:// doi.org/10.1093/nar/gkq603

**Table S1** The sequencing data of HiFi in *Neolamarckia macrophylla*

| Type | Size |
| --- | --- |
| Sequencing depth | 67 × |
| Total number of reads | 2,176,900 |
| Base number of sequencing | 37,332,239,871 bp |
| Reads length N50 | 17,329 bp |
| Reads length mean | 17,149 bp |
| Reads length max | 55,092 bp |

**Table S2** The sequencing data of Hi-C in *Neolamarckia macrophylla*

| Type | Size |
| --- | --- |
| Total number of reads | 264,084,028 |
| Base number of sequencing | 79,036,071,208 bp |
| GC (%) | 36.56 |
| N (%) | 0.01 |
| Q20 (%) | 98.17 |
| Q30 (%) | 94.92 |

**Table S3** The genome assembly data from Hi-C analysis in *Neolamarckia macrophylla*

| Group | Cluster number | Cluster length (bp) | Order number | Order length (bp) |
| --- | --- | --- | --- | --- |
| chr01 | 3 | 36,052,163 | 2 | 36,004,432 |
| chr02 | 3 | 31,333,304 | 1 | 31,134,050 |
| chr03 | 7 | 36,000,864 | 1 | 35,559,994 |
| chr04 | 11 | 29,770,344 | 1 | 28,336,096 |
| chr05 | 7 | 31,814,312 | 1 | 31,179,135 |
| chr06 | 5 | 26,166,413 | 1 | 25,766,800 |
| chr07 | 6 | 22,524,259 | 1 | 22,067,796 |
| chr08 | 5 | 27,744,972 | 1 | 27,207,025 |
| chr09 | 5 | 24,756,944 | 1 | 23,991,271 |
| chr10 | 4 | 23,111,613 | 1 | 22,888,008 |
| chr11 | 3 | 25,081,290 | 1 | 24,935,073 |
| chr12 | 9 | 25,284,537 | 3 | 24,564,038 |
| chr13 | 6 | 24,399,097 | 3 | 24,134,716 |
| chr14 | 5 | 23,694,760 | 3 | 23,504,842 |
| chr15 | 6 | 24,905,506 | 1 | 24,540,723 |
| chr16 | 6 | 23,740,080 | 1 | 23,405,752 |
| chr17 | 8 | 22,866,711 | 1 | 22,275,422 |
| chr18 | 7 | 22,323,151 | 1 | 21,348,825 |
| chr19 | 4 | 21,046,991 | 1 | 20,818,997 |
| chr20 | 4 | 25,021,145 | 2 | 24,710,024 |
| chr21 | 4 | 21,265,605 | 1 | 21,057,312 |
| chr22 | 10 | 19,528,330 | 1 | 18,883,641 |
| Total  (Ratio %) | 128(58.72) | 568,432,391(94.99) | 30(23.44) | 558,313,972(98.22) |

**Table S4** Gene annotation based on different databases from sequencing of *Neolamarckia macrophylla* genome

| Database for annotation | Annotated number | Annotated ratio（%） |
| --- | --- | --- |
| GO | 28,337 | 82.35 |
| KEGG | 25,121 | 73.00 |
| KOG | 18,593 | 54.03 |
| Pfam | 29,512 | 85.76 |
| Swissprot | 26,008 | 75.58 |
| TrEMBL | 3,440 | 97.18 |
| eggNOG | 28,753 | 83.56 |
| Nr | 33,048 | 96.04 |
| All annotated | 33,620 | 97.70 |

**Table S5** Annotations of genes under strong purifying and positive selection in expanded gene families

| Species | ID | *K_a_/K_s_* | Function |
| --- | --- | --- | --- |
| *N. macrophylla* | *Nma08G013120.1* | 0.0325 | 2-OGDD19, involved in caffeine biosynthesis pathway |
|  | *Nma05G006810.1* | 0.0546 | Strictosidine synthase, key Enzymes for monoterpenoid indole alkaloid biosynthesis |
|  | *Nma19G006250.1* | 0.105 | Uncharacterized protein |
|  | *Nma05G001020.1* | 0.109 | ACC oxidase catalyzes the terminal step of ethylene biosynthesis |
|  | *Nma09G003940.1* | 0.109 | Receptor-like kinases mediating disease resistance responses |
|  | *Nma22G009720.1* | 4.30353 | Uncharacterized protein |
|  |  |  |  |
| *N. cadamba* | *evm.model.Contig271.116* | 0.0928876 | Dual-specificity phosphatase 4 (DSP4) |
|  | *evm.model.Contig12.291* | 0.10394 | Detoxification efflux carrier 27 (DETOXIFICATION 27) |
|  | *evm.model.Contig271.112* | 0.109279 | 1-Aminocyclopropane-1-carboxylate oxidase (ACO) |
|  | *evm.model.Contig302* | 1.02 | Uncharacterized protein |

**Table S6** Phenotypic variation of wood traits in two samples of *N. cadamba* for re-sequencing

| Trait* | Age | Range | Mean ± SD | CV(%) | *P*-Value^#^ |
| --- | --- | --- | --- | --- | --- |
| FL (μm) | 7 | 1072.86~1699.10 | 1411.38±139.50 | 9.88 | 0.0809 |
|  | 12 | 1133.03~1848.42 | 1539.06±158.58 | 10.30 | 0.1906 |
| FD (μm) | 7 | 28.45~35.76 | 32.49±1.42 | 4.38 | 0.1836 |
|  | 12 | 25.80~34.50 | 29.28±1.43 | 4.88 | 0.0110 |
| VL (μm) | 7 | 491.42~858.10 | 663.17±77.06 | 11.62 | 0.0363 |
|  | 12 | 351.58~768.29 | 526.98±78.06 | 14.81 | 0.6575 |
| VD (μm) | 7 | 99.36~234.43 | 158.01±27.10 | 17.15 | 0.2622 |
|  | 12 | 125.37~221.71 | 169.86±18.34 | 10.80 | 0.9626 |
| BD (g/cm³) | 7 | 0.26~0.40 | 0.335±0.03 | 9.25 | 0.2815 |
|  | 12 | 0.25~0.39 | 0.316±0.03 | 9.49 | 0.8797 |
| FL/FD | 7 | 36.42~51.13 | 43.41±3.43 | 7.91 | 0.2586 |
|  | 12 | 38.80~64.51 | 52.65±5.77 | 10.96 | 0.5742 |
| VL/VD | 7 | 2.95~6.81 | 4.29±0.72 | 16.85 | 0.0463 |
|  | 12 | 2.27~3.91 | 3.11±0.35 | 11.25 | 0.4029 |

*: FL: fiber length; FD: fiber diameter; VL: vessel length; VD: vessel diameter; BD: basic density (BD).

#: *P*-value: normal distribution test with Kolmogorov-Smirnov (K-S) method in R.

**Table S7** Correlations among wood property traits for the first sample (age 7) of *N. cadamba*#

| Trait | FL | FD | VL | VD | BD | FL/FD | VL/VD | |
| --- | --- | --- | --- | --- | --- | --- | --- | --- |
| FL | 1 |  |  |  |  |  |  |  |
| FD | 0.638*** | 1 |  |  |  |  |  | |
| VL | 0.568*** | 0.430*** | 1 |  |  |  |  | |
| VD | 0.708*** | 0.692*** | 0.373*** | 1 |  |  |  | |
| BD | -0.236* | -0.406** | -0.311** | -0.273** | 1 |  |  | |
| FL/FD | 0.912*** | 0.267** | 0.474*** | 0.516*** | -0.079 | 1 |  | |
| VL/VD | -0.377*** | -0.430*** | 0.290** | -0.757*** | 0.100 | -0.248* | 1 | |

#: FL: fiber length; FD: fiber diameter; VL: vessel length; VD: vessel diameter; BD: basic density (BD).

*: *P*-value < 0.05; **: *P*-value < 0.01; ***: *P*-value < 0.001.

**Table S8** Correlations among wood property traits for the second sample (age 12) of *N. cadamba* #

| Trait | FL | FD | VL | VD | BD | FL/FD | VL/VD |
| --- | --- | --- | --- | --- | --- | --- | --- |
| FL | 1 |  |  |  |  |  |  |
| FD | 0.10 | 1 |  |  |  |  |  |
| VL | 0.72^***^ | 0.01 | 1 |  |  |  |  |
| VD | 0.60^***^ | 0.04 | 0.67^***^ | 1 |  |  |  |
| BD | -0.02 | -0.12 | -0.07 | -0.09 | 1 |  |  |
| FL/FD | 0.90^***^ | -0.34^***^ | 0.67^***^ | 0.55^***^ | 0.04 | 1 |  |
| VL/VD | 0.36^***^ | -0.04 | 0.67 | -0.11 | 0 | 0.35^***^ | 1 |

#: FL: fiber length; FD: fiber diameter; VL: vessel length; VD: vessel diameter; BD: basic density (BD).

*: *P*-value < 0.05; **: *P*-value < 0.01; ***: *P*-value < 0.001.

**Table S9** Distribution of SNPs across chromosomes in the first sample (age 7) of *N. cadamba*

| Chromosome | SNP coverage length (bp) | Number  of SNPs | SNP density （bp/SNP） | Percentage of SNPs (%) |
| --- | --- | --- | --- | --- |
| Chr01 | 40,167,398 | 373,020 | 107.68 | 6.63 |
| Chr02 | 36,798,086 | 277,177 | 132.76 | 4.93 |
| Chr03 | 35,147,831 | 318,718 | 110.28 | 5.67 |
| Chr04 | 34,714,966 | 327,384 | 106.04 | 5.82 |
| Chr05 | 34,116,711 | 276,197 | 123.52 | 4.91 |
| Chr06 | 33,796,703 | 255,862 | 132.09 | 4.55 |
| Chr07 | 33,675,061 | 218,839 | 153.88 | 3.89 |
| Chr08 | 32,439,964 | 284,681 | 113.95 | 5.06 |
| Chr09 | 31,407,371 | 234,761 | 133.78 | 4.17 |
| Chr10 | 29,334,823 | 229,397 | 127.88 | 4.08 |
| Chr11 | 29,201,329 | 303,694 | 96.15 | 5.40 |
| Chr12 | 29,144,692 | 237,995 | 122.46 | 4.23 |
| Chr13 | 28,929,231 | 220,986 | 130.91 | 3.93 |
| Chr14 | 28,548,636 | 247,710 | 115.25 | 4.40 |
| Chr15 | 27,537,281 | 265,080 | 103.88 | 4.71 |
| Chr16 | 27,217,201 | 258,211 | 105.41 | 4.59 |
| Chr17 | 27,041,525 | 220,820 | 122.46 | 3.93 |
| Chr18 | 27,025,816 | 250,309 | 107.97 | 4.45 |
| Chr19 | 26,396,942 | 198,940 | 132.69 | 3.54 |
| Chr20 | 25,679,085 | 207,154 | 123.96 | 3.68 |
| Chr21 | 25,386,097 | 191,989 | 132.23 | 3.41 |
| Chr22 | 24,501,539 | 224,505 | 109.14 | 3.99 |
| ALL | 668,208,288 | 5,623,429 | 118.83 | 100.00 |

**Table S10** Distribution of SNPs across chromosomes in the second sample (age 12) of *N. cadamba*

| Chromosome | SNP coverage length (bp) | Number  of SNPs | SNP Density （bp/SNP） | Percentage  of SNPs (%) |
| --- | --- | --- | --- | --- |
| Chr01 | 40,165,801 | 470,311 | 85.4 | 6.54 |
| Chr02 | 36,798,169 | 356,255 | 103.3 | 4.95 |
| Chr03 | 35,145,040 | 402,115 | 87.4 | 5.59 |
| Chr04 | 34,729,358 | 439,677 | 79.0 | 6.11 |
| Chr05 | 34,118,231 | 348,642 | 97.9 | 4.85 |
| Chr06 | 33,796,021 | 327,115 | 103.3 | 4.55 |
| Chr07 | 33,677,018 | 313,977 | 107.3 | 4.37 |
| Chr08 | 32,439,786 | 360,249 | 90.0 | 5.01 |
| Chr09 | 31,418,724 | 291,029 | 108.0 | 4.05 |
| Chr10 | 29,348,549 | 293,042 | 100.2 | 4.08 |
| Chr11 | 29,199,064 | 372,149 | 78.5 | 5.18 |
| Chr12 | 29,158,828 | 315,114 | 92.5 | 4.38 |
| Chr13 | 28,917,997 | 269,435 | 107.3 | 3.75 |
| Chr14 | 28,547,854 | 310,240 | 92.0 | 4.31 |
| Chr15 | 27,537,784 | 328,346 | 83.9 | 4.57 |
| Chr16 | 27,213,618 | 311,035 | 87.5 | 4.33 |
| Chr17 | 27,040,070 | 297,899 | 90.8 | 4.14 |
| Chr18 | 27,026,014 | 311,669 | 86.7 | 4.33 |
| Chr19 | 26,397,814 | 255,094 | 103.5 | 3.55 |
| Chr20 | 25,686,874 | 266,462 | 96.4 | 3.71 |
| Chr21 | 25,387,949 | 250,424 | 101.4 | 3.48 |
| Chr22 | 24,503,980 | 300,632 | 81.5 | 4.18 |
| Total | 668,254,543 | 7,190,911 | 92.9 | 100.00 |

**Table S11** Distribution of SNPs under Hardy-Weinberg disequilibrium (HWD) across chromosomes in two samples of *N. cadamba*

| Chromosome | 1^st^ sample  Number of SNPs | SNPs with HWD (%) | 2^nd^ sample  Number of SNPs | SNPs with HWD (%) |
| --- | --- | --- | --- | --- |
| Chr01 | 33,122 | 7.46 | 47,715 | 6.27 |
| Chr02 | 18,530 | 4.17 | 28,945 | 3.80 |
| Chr03 | 20,765 | 4.68 | 39,105 | 5.14 |
| Chr04 | 29,101 | 6.55 | 57,493 | 7.55 |
| Chr05 | 17,261 | 3.89 | 30,335 | 3.98 |
| Chr06 | 24,029 | 5.41 | 35,454 | 4.66 |
| Chr07 | 14,511 | 3.27 | 26,871 | 3.53 |
| Chr08 | 28,402 | 6.40 | 36,991 | 4.86 |
| Chr09 | 18,232 | 4.11 | 32,589 | 4.28 |
| Chr10 | 18,192 | 4.10 | 37,158 | 4.88 |
| Chr11 | 14,792 | 3.33 | 36,692 | 4.82 |
| Chr12 | 18,229 | 4.10 | 35,863 | 4.71 |
| Chr13 | 12,763 | 2.87 | 21,731 | 2.85 |
| Chr14 | 16,186 | 3.64 | 25,294 | 3.32 |
| Chr15 | 14,144 | 3.19 | 22,688 | 2.98 |
| Chr16 | 26,089 | 5.87 | 40,985 | 5.38 |
| Chr17 | 34,715 | 7.82 | 47,203 | 6.20 |
| Chr18 | 22,870 | 5.15 | 39,417 | 5.18 |
| Chr19 | 17,475 | 3.94 | 29,985 | 3.94 |
| Chr20 | 16,366 | 3.69 | 27,886 | 3.66 |
| Chr21 | 12,180 | 2.74 | 25,069 | 3.29 |
| Chr22 | 16,115 | 3.63 | 35,879 | 4.71 |

**Table S12**. Chromosomal positions of SNPs associated with wood property traits from GWAS of *N*. *cadamba*

|  | Wood traits | Chromosome | Position | p-value |
| --- | --- | --- | --- | --- |
| The first sample (age 7) | FL | chr15 | 2404139 | 4.650781e-07 |
|  |  |  |  |  |
|  | FD | chr21 | 5848411 | 4.583306e-07 |
|  |  | chr21 | 5846303 | 4.936020e-07 |
|  |  | chr21 | 5850623 | 5.019433e-07 |
|  |  |  |  |  |
|  | VL | chr10 | 28997993 | 3.917188e-07 |
|  |  |  |  |  |
|  | VD | chr19 | 11078179 | 3.144060e-09 |
|  |  | chr19 | 11065556 | 3.544609e-09 |
|  |  | chr19 | 11077060 | 4.633145e-09 |
|  |  | chr19 | 11067727 | 5.285714e-09 |
|  |  | chr19 | 11067734 | 5.285714e-09 |
|  |  | chr19 | 11080877 | 7.406205e-09 |
|  |  | chr19 | 11079683 | 7.987833e-09 |
|  |  | chr19 | 11085847 | 8.466852e-09 |
|  |  | chr19 | 11087161 | 1.858008e-08 |
|  |  | chr19 | 11079130 | 1.921643e-08 |
|  |  | chr19 | 11067892 | 1.934180e-08 |
|  |  | chr19 | 11074768 | 2.270736e-08 |
|  |  | chr19 | 11076154 | 2.287390e-08 |
|  |  | chr19 | 11078038 | 2.287390e-08 |
|  |  | chr19 | 11084012 | 2.320433e-08 |
|  |  | chr19 | 11087119 | 2.345594e-08 |
|  |  | chr19 | 11068515 | 2.374722e-08 |
|  |  | chr19 | 11075822 | 2.477916e-08 |
|  |  | chr19 | 11086104 | 2.677351e-08 |
|  |  | chr19 | 11086098 | 2.692273e-08 |
|  |  | chr19 | 11080442 | 2.727404e-08 |
|  |  | chr19 | 11073559 | 2.811830e-08 |
|  |  | chr19 | 11067453 | 2.837032e-08 |
|  |  | chr19 | 11067801 | 2.837032e-08 |
|  |  | chr19 | 11068550 | 2.837032e-08 |
|  |  | chr19 | 11068658 | 2.837032e-08 |
|  |  | chr19 | 11075384 | 2.837032e-08 |
|  |  | chr19 | 11075912 | 2.837032e-08 |
|  |  | chr19 | 11076305 | 2.837032e-08 |
|  |  | chr19 | 11076308 | 2.837032e-08 |
|  |  | chr19 | 11077323 | 2.837032e-08 |
|  |  | chr19 | 11078066 | 2.837032e-08 |
|  |  | chr19 | 11078067 | 2.837032e-08 |
|  |  | chr19 | 11079233 | 2.837032e-08 |
|  |  | chr19 | 11079243 | 2.837032e-08 |
|  |  | chr19 | 11079275 | 2.837032e-08 |
|  |  | chr19 | 11079846 | 2.837032e-08 |
|  |  | chr19 | 11081397 | 2.837032e-08 |
|  |  | chr19 | 11083057 | 2.837032e-08 |
|  |  | chr19 | 11086279 | 2.837032e-08 |
|  |  | chr19 | 24604422 | 2.841362e-08 |
|  |  | chr19 | 11080304 | 2.853342e-08 |
|  |  | chr19 | 11077266 | 2.972843e-08 |
|  |  | chr19 | 24604435 | 2.984363e-08 |
|  |  | chr19 | 11083369 | 3.032832e-08 |
|  |  | chr19 | 11078749 | 3.035040e-08 |
|  |  | chr19 | 11086881 | 3.045343e-08 |
|  |  | chr06 | 32506082 | 3.049373e-08 |
|  |  | chr19 | 11073637 | 3.099551e-08 |
|  |  | chr19 | 11067434 | 3.140712e-08 |
|  |  | chr19 | 11074935 | 3.140712e-08 |
|  |  | chr19 | 11068438 | 3.149358e-08 |
|  |  | chr19 | 11067079 | 3.171476e-08 |
|  |  | chr19 | 11085660 | 3.242169e-08 |
|  |  | chr19 | 11078788 | 3.359060e-08 |
|  |  | chr19 | 11067544 | 3.378124e-08 |
|  |  | chr19 | 11067558 | 3.378124e-08 |
|  |  | chr19 | 11078608 | 3.378124e-08 |
|  |  | chr19 | 11080236 | 3.378124e-08 |
|  |  | chr19 | 11080266 | 3.378124e-08 |
|  |  | chr19 | 11077096 | 3.434547e-08 |
|  |  | chr19 | 11079027 | 3.452308e-08 |
|  |  | chr19 | 11081533 | 3.558717e-08 |
|  |  | chr19 | 11081535 | 3.558717e-08 |
|  |  | chr19 | 11079056 | 3.568026e-08 |
|  |  | chr19 | 11066561 | 3.632163e-08 |
|  |  | chr19 | 11081719 | 3.693898e-08 |
|  |  | chr19 | 11083446 | 3.888581e-08 |
|  |  | chr19 | 11075153 | 3.889889e-08 |
|  |  | chr19 | 11078705 | 4.035681e-08 |
|  |  | chr19 | 11079794 | 4.118609e-08 |
|  |  | chr19 | 11074046 | 4.138873e-08 |
|  |  | chr19 | 11067137 | 4.266927e-08 |
|  |  | chr19 | 11073782 | 4.266927e-08 |
|  |  | chr19 | 11078147 | 4.401057e-08 |
|  |  | chr19 | 11065612 | 4.451918e-08 |
|  |  | chr19 | 11061515 | 4.472109e-08 |
|  |  | chr19 | 11068309 | 4.472109e-08 |
|  |  | chr19 | 11083587 | 4.802670e-08 |
|  |  | chr19 | 11068771 | 4.832578e-08 |
|  |  | chr19 | 11079753 | 4.964607e-08 |
|  |  | chr19 | 11066626 | 5.234412e-08 |
|  |  | chr19 | 11081667 | 5.339047e-08 |
|  |  | chr19 | 11081677 | 5.339047e-08 |
|  |  | chr19 | 11085450 | 5.463823e-08 |
|  |  | chr19 | 11085342 | 5.495593e-08 |
|  |  | chr19 | 11068733 | 5.927413e-08 |
|  |  | chr19 | 11075224 | 5.958046e-08 |
|  |  | chr19 | 11073426 | 6.320526e-08 |
|  |  | chr19 | 11086619 | 6.681295e-08 |
|  |  | chr19 | 11076669 | 6.741060e-08 |
|  |  | chr19 | 11076713 | 6.741060e-08 |
|  |  | chr19 | 11073618 | 6.901423e-08 |
|  |  | chr19 | 11075245 | 7.034245e-08 |
|  |  | chr19 | 11074271 | 7.397398e-08 |
|  |  | chr19 | 11081786 | 7.565785e-08 |
|  |  | chr19 | 11073479 | 7.849064e-08 |
|  |  | chr19 | 11066476 | 7.947083e-08 |
|  |  | chr19 | 11081142 | 8.182406e-08 |
|  |  | chr06 | 32508113 | 8.735519e-08 |
|  |  | chr19 | 11081784 | 8.953277e-08 |
|  |  | chr19 | 11077798 | 9.009008e-08 |
|  |  | chr19 | 11082222 | 9.324535e-08 |
|  |  | chr06 | 32507414 | 1.024142e-07 |
|  |  | chr19 | 11083869 | 1.065845e-07 |
|  |  | chr19 | 11076367 | 1.205148e-07 |
|  |  | chr19 | 11081954 | 1.260086e-07 |
|  |  | chr06 | 32506644 | 1.297960e-07 |
|  |  | chr19 | 24605141 | 1.361678e-07 |
|  |  | chr19 | 11083366 | 1.423666e-07 |
|  |  | chr19 | 11085057 | 1.426544e-07 |
|  |  | chr19 | 11085175 | 1.596577e-07 |
|  |  | chr19 | 11074943 | 1.607518e-07 |
|  |  | chr06 | 32506685 | 1.661222e-07 |
|  |  | chr06 | 32505897 | 1.748577e-07 |
|  |  | chr19 | 11079173 | 1.849209e-07 |
|  |  | chr19 | 24601467 | 1.899412e-07 |
|  |  | chr19 | 11086605 | 2.048598e-07 |
|  |  | chr06 | 32506892 | 2.187624e-07 |
|  |  | chr19 | 11074113 | 2.234994e-07 |
|  |  | chr19 | 11082893 | 2.236297e-07 |
|  |  | chr06 | 32506962 | 2.261983e-07 |
|  |  | chr19 | 11080000 | 2.335514e-07 |
|  |  | chr04 | 351031 | 2.346485e-07 |
|  |  | chr19 | 11076585 | 2.369726e-07 |
|  |  | chr19 | 24606421 | 2.416091e-07 |
|  |  | chr19 | 11076797 | 2.435666e-07 |
|  |  | chr19 | 11083053 | 2.442522e-07 |
|  |  | chr19 | 11074278 | 2.450265e-07 |
|  |  | chr06 | 32506436 | 2.454501e-07 |
|  |  | chr04 | 380330 | 2.460808e-07 |
|  |  | chr04 | 380336 | 2.460808e-07 |
|  |  | chr19 | 11077255 | 2.531841e-07 |
|  |  | chr19 | 11068066 | 2.705624e-07 |
|  |  | chr19 | 24608985 | 2.731447e-07 |
|  |  | chr04 | 380335 | 2.742078e-07 |
|  |  | chr19 | 11075962 | 2.928843e-07 |
|  |  | chr19 | 24605318 | 3.019266e-07 |
|  |  | chr19 | 11081295 | 3.144615e-07 |
|  |  | chr06 | 29034217 | 3.302570e-07 |
|  |  | chr19 | 24597096 | 3.409676e-07 |
|  |  | chr19 | 11079655 | 3.557727e-07 |
|  |  | chr19 | 11083526 | 3.574304e-07 |
|  |  | chr19 | 11083525 | 3.853738e-07 |
|  |  | chr05 | 2217466 | 3.988775e-07 |
|  |  | chr19 | 11083150 | 3.991817e-07 |
|  |  | chr04 | 188798 | 4.023786e-07 |
|  |  | chr04 | 122295 | 4.027710e-07 |
|  |  | chr06 | 32506262 | 4.073777e-07 |
|  |  | chr19 | 24597105 | 4.359108e-07 |
|  |  |  |  |  |
|  | BD | chr19 | 12496876 | 1.721773e-08 |
|  |  | chr19 | 12432456 | 3.331977e-08 |
|  |  | chr19 | 12634808 | 8.553273e-08 |
|  |  | chr19 | 12637900 | 8.704708e-08 |
|  |  | chr19 | 12268119 | 9.870727e-08 |
|  |  | chr19 | 12465737 | 9.885257e-08 |
|  |  | chr19 | 12728802 | 1.033966e-07 |
|  |  | chr03 | 9878370 | 1.150254e-07 |
|  |  | chr19 | 12579563 | 1.209576e-07 |
|  |  | chr19 | 12518192 | 1.284267e-07 |
|  |  | chr19 | 12521701 | 1.284267e-07 |
|  |  | chr19 | 12548824 | 1.284267e-07 |
|  |  | chr19 | 12579769 | 1.284267e-07 |
|  |  | chr19 | 12607464 | 1.284267e-07 |
|  |  | chr19 | 12672888 | 1.284267e-07 |
|  |  | chr19 | 12732578 | 1.284267e-07 |
|  |  | chr19 | 12758085 | 1.359561e-07 |
|  |  | chr03 | 9907678 | 1.439307e-07 |
|  |  | chr19 | 12752839 | 1.565629e-07 |
|  |  | chr03 | 9877077 | 1.751133e-07 |
|  |  | chr19 | 12442083 | 1.869155e-07 |
|  |  | chr19 | 12565939 | 2.080965e-07 |
|  |  | chr19 | 12258965 | 2.112905e-07 |
|  |  | chr19 | 12453769 | 2.116834e-07 |
|  |  | chr19 | 12641471 | 2.156735e-07 |
|  |  | chr19 | 12641477 | 2.156735e-07 |
|  |  | chr19 | 12221520 | 2.218362e-07 |
|  |  | chr19 | 12442717 | 2.334306e-07 |
|  |  | chr19 | 12550101 | 2.634449e-07 |
|  |  | chr19 | 12594818 | 2.760327e-07 |
|  |  | chr19 | 12634482 | 2.760327e-07 |
|  |  | chr19 | 12638682 | 2.760327e-07 |
|  |  | chr19 | 12655612 | 2.760327e-07 |
|  |  | chr19 | 12689253 | 2.760327e-07 |
|  |  | chr19 | 12736182 | 2.760327e-07 |
|  |  | chr19 | 12737524 | 2.760327e-07 |
|  |  | chr19 | 12767901 | 2.760327e-07 |
|  |  | chr19 | 12783132 | 2.760327e-07 |
|  |  | chr19 | 12789316 | 2.760327e-07 |
|  |  | chr03 | 9908152 | 2.935636e-07 |
|  |  | chr19 | 12594443 | 2.950525e-07 |
|  |  | chr19 | 12594444 | 2.950525e-07 |
|  |  | chr19 | 12781364 | 3.094517e-07 |
|  |  | chr19 | 12565190 | 3.208277e-07 |
|  |  | chr19 | 12440346 | 3.574546e-07 |
|  |  | chr03 | 9907443 | 3.605197e-07 |
|  |  | chr19 | 12425059 | 3.653716e-07 |
|  |  | chr19 | 12407403 | 3.671164e-07 |
|  |  | chr19 | 12691235 | 4.106712e-07 |
|  |  | chr03 | 9881508 | 4.132440e-07 |
|  |  | chr19 | 12431540 | 4.206063e-07 |
|  |  | chr19 | 12799009 | 4.351234e-07 |
|  |  | chr19 | 12513346 | 4.443398e-07 |
|  |  | chr03 | 9877204 | 4.536909e-07 |
|  |  | chr19 | 12085710 | 4.694631e-07 |
|  |  | chr19 | 12481713 | 4.873688e-07 |
|  |  | chr03 | 9881935 | 4.873949e-07 |
|  |  | chr19 | 12240158 | 4.913122e-07 |
|  |  | chr03 | 9880550 | 4.945298e-07 |
|  |  |  |  |  |
|  | VL/VD | chr01 | 33454649 | 1.449480e-08 |
|  |  | chr15 | 16835862 | 2.864474e-08 |
|  |  | chr15 | 16823696 | 5.379375e-08 |
|  |  | chr15 | 16942644 | 7.531757e-08 |
|  |  | chr15 | 16914008 | 1.519563e-07 |
|  |  | chr15 | 16864218 | 1.806681e-07 |
|  |  | chr15 | 16976841 | 1.879503e-07 |
|  |  | chr15 | 16923695 | 2.113946e-07 |
|  |  | chr15 | 16663649 | 2.238403e-07 |
|  |  | chr15 | 16901901 | 2.238403e-07 |
|  |  | chr15 | 16921376 | 2.238403e-07 |
|  |  | chr15 | 16950365 | 2.238403e-07 |
|  |  | chr15 | 16953237 | 2.238403e-07 |
|  |  | chr07 | 24703076 | 2.587510e-07 |
|  |  | chr15 | 16821704 | 3.218122e-07 |
|  |  | chr12 | 11008845 | 4.391364e-07 |
|  |  |  |  |  |
| The second sample (age 12) | FD | chr20 | 17634027 | 7.620741e-08 |
|  |  | chr11 | 13287624 | 8.268782e-08 |
|  |  | chr19 | 18231979 | 1.141242e-07 |
|  |  | chr11 | 14195852 | 1.740887e-07 |
|  |  |  |  |  |
|  | VD | chr17 | 3824776 | 1.663761e-07 |
|  |  | chr17 | 3825173 | 1.663761e-07 |
|  |  |  |  |  |
|  | BD | chr01 | 31547953 | 1.440937e-07 |
|  |  |  |  |  |
|  | FL/FD | chr15 | 21034613 | 6.232408e-08 |

**Table S13** Annotations of candidate genes covering the SNPs associated with wood traits within 50 kb in two samples of *N. cadamba*

| Sample | Trait | SNP position | Gene ID | Candidate gene function |
| --- | --- | --- | --- | --- |
| 1^st^ | FL | chr15_2404139 | *evm.model.Contig381.45* | GTP-binding protein BRASSINAZOLE INSENSITIVE PALE GREEN 2 (BPG2) |
|  |  |  | *evm.model.Contig381.46* | WEB family protein, chloroplastic-like isoform X1 |
|  |  |  | *evm.model.Contig381.47* | unnamed protein product |
|  |  |  | *evm.model.Contig381.48* | casein kinase 1-like protein HD16 (HD16) |
|  |  |  |  |  |
|  | FD | chr21_5848411 | *evm.model.Contig143.104* | UDP-glycosyltransferase 89B2-like |
|  |  | chr21_5846303 | *evm.model.Contig143.105* | unnamed protein product |
|  |  | chr21_5850623 | *evm.model.Contig143.106* | protein ENHANCER OF LHP1 1-like |
|  |  |  | *evm.model.Contig143.109* | tropinone reductase homolog At2g29340-like |
|  |  |  | *evm.model.Contig143.110* | senescence-associated protein 13-like |
|  |  |  | *evm.model.Contig143.111* | tropinone reductase homolog At5g06060-like |
|  |  |  | *evm.model.Contig143.112* | unnamed protein product |
|  |  |  | *evm.model.Contig143.114* | uncharacterized protein |
|  |  |  |  |  |
|  | VL | chr10_28997993 | *evm.model.Contig480.18* | uncharacterized protein |
|  |  |  | *evm.model.Contig480.19* | histidine--tRNA ligase, cytoplasmic |
|  |  |  |  |  |
|  | VD | chr19_11078179 | *evm.model.Contig1.24* | probable RNA-dependent RNA polymerase 1 |
|  |  | chr19_11065556 | *evm.model.Contig1.25* | methyl-CpG-binding domain-containing protein 4-like |
|  |  | chr19_11077060 | *evm.model.Contig1.26.1* | pre-mRNA-splicing factor ATP-dependent RNA helicase DEAH7 isoform X1 |
|  |  | chr19_11067727 | *evm.model.Contig1.27* | two-component response regulator ORR21 |
|  |  | chr19_11067734 | *evm.model.Contig1.28* | uncharacterized protein |
|  |  | chr19_11080877 | *evm.model.Contig1.29* | uncharacterized protein |
|  |  | chr19_11079683 | *evm.model.Contig154.174* | protein PHYLLO, chloroplastic isoform X3 |
|  |  | chr19_11085847 | *evm.model.Contig154.175* | histidine--tRNA ligase, cytoplasmic |
|  |  | chr19_11087161 | *evm.model.Contig154.176* | unnamed protein product |
|  |  | chr19_11079130 | *evm.model.Contig154.177_evm.model.Contig154.178* | endoribonuclease Dicer homolog 1 isoform X1 |
|  |  | chr19_11067892 | *evm.model.Contig154.179* | protein LHY |
|  |  | chr19_11074768 | *evm.model.Contig154.180* | unnamed protein product |
|  |  | chr19_11076154 | *evm.model.Contig154.181* | unnamed protein product |
|  |  | chr19_11078038 | *evm.model.Contig154.182* | vacuolar protein sorting-associated protein 8 homolog isoform X1 |
|  |  | chr19_11084012 | *evm.model.Contig154.183* | vacuolar protein sorting-associated protein 8 homolog isoform X1 |
|  |  | chr19_11087119 | *evm.model.Contig154.184* | vacuolar protein sorting-associated protein 8 homolog isoform X1 |
|  |  | chr19_11068515 | *evm.model.Contig154.185* | uncharacterized protein |
|  |  | chr19_11075822 | *evm.model.Contig35.60* | calcium-binding protein PBP1-like |
|  |  | chr19_11086104 | *evm.model.Contig35.61* | unnamed protein product |
|  |  | chr19_11086098 | *evm.model.Contig35.62* | unnamed protein product |
|  |  | chr19_11080442 | *evm.model.Contig35.63* | vacuolar import and degradation protein 30-like isoform X3 |
|  |  | chr19_11073559 | *evm.model.Contig35.64* | universal stress protein PHOS32 isoform X1 |
|  |  | chr19_11067453 | *evm.model.Contig35.65* | uncharacterized protein |
|  |  | chr19_11067801 | *evm.model.Contig35.66* | uncharacterized protein |
|  |  | chr19_11068550 | *evm.model.Contig35.67* | transcription factor MYB57-like |
|  |  | chr19_11068658 | *evm.model.Contig35.68* | disease resistance RPP13-like protein 4 |
|  |  | chr19_11075384 | *evm.model.Contig35.69* | probable disease resistance protein At5g45490 |
|  |  | chr19_11075912 | *evm.model.Contig35.70* | uncharacterized protein |
|  |  | chr19_11076305 | *evm.model.Contig35.71* | unnamed protein product |
|  |  | chr19_11076308 | *evm.model.Contig35.72* | uncharacterized protein |
|  |  | chr19_11077323 | *evm.model.Contig35.73* | equilibrative nucleotide transporter 3-like |
|  |  | chr19_11078066 | *evm.model.Contig437.73* | probable sucrose-phosphate synthase 1（SPS1） |
|  |  | chr19_11078067 | *evm.model.Contig437.74* | unnamed protein product |
|  |  | chr19_11079233 | *evm.model.Contig437.75* | probable glycosyltransferase At5g25310 |
|  |  | chr19_11079243 | *evm.model.Contig437.76* | unnamed protein product |
|  |  | chr19_11079275 | *evm.model.Contig437.77* | unnamed protein product |
|  |  | chr19_11079846 | *evm.model.Contig437.78* | protein FAR1-RELATED SEQUENCE 6 isoform X1 |
|  |  | chr19_11081397 | *evm.model.Contig437.79* | vesicle-associated membrane protein 711 isoform X1 |
|  |  | chr19_11083057 | *evm.model.Contig437.80* | unnamed protein product |
|  |  | chr19_11086279 | *evm.model.Contig437.81* | unnamed protein product |
|  |  | chr19_24604422 | *evm.model.Contig437.82* | unnamed protein product |
|  |  | chr19_11080304 | *evm.model.Contig437.83* | unnamed protein product |
|  |  | chr19_11077266 | *evm.model.Contig437.84* | E3 ubiquitin-protein ligase KEG |
|  |  | chr19_24604435 | *evm.model.Contig437.86* | uncharacterized protein |
|  |  | chr19_11083369 | *evm.model.Contig437.87* | gibberellin 2-beta-dioxygenase 2 |
|  |  | chr19_11078749 | *evm.model.Contig7.100* | myosin-1 isoform X1 |
|  |  | chr19_11086881 | *evm.model.Contig7.101* | methylenetetrahydrofolate reductase 2-like |
|  |  | chr6_32506082 | *evm.model.Contig7.102* | methylenetetrahydrofolate reductase 2-like |
|  |  | chr19_11073637 | *evm.model.Contig7.311* | pentatricopeptide repeat-containing protein At5g02860-like isoform |
|  |  | chr19_11067434 | *evm.model.Contig7.312* | unnamed protein product |
|  |  | chr19_11074935 | *evm.model.Contig7.313* | rhomboid-like protein 11, chloroplastic |
|  |  | chr19_11068438 | *evm.model.Contig7.314* | sulfite exporter TauE/SafE family protein 3 |
|  |  | chr19_11067079 | *evm.model.Contig7.315* | dirigent protein 25-like (DIR25) |
|  |  | chr19_11085660 | *evm.model.Contig7.316* | unnamed protein product |
|  |  | chr19_11078788 | *evm.model.Contig7.317* | Coiled-coil domain-containing protein 25 |
|  |  | chr19_11067544 | *evm.model.Contig7.318* | la protein 1 |
|  |  | chr19_11067558 | *evm.model.Contig7.319* | unnamed protein product |
|  |  | chr19_11078608 | *evm.model.Contig7.320* | aspartate aminotransferase, cytoplasmic |
|  |  | chr19_11080236 | *evm.model.Contig7.94.1* | uncharacterized protein |
|  |  | chr19_11080266 | *evm.model.Contig7.95* | zinc finger CCCH domain-containing protein 48-like isoform X3 |
|  |  | chr19_11077096 | *evm.model.Contig7.96* | uncharacterized protein |
|  |  | chr19_11079027 | *evm.model.Contig7.99* | protein-S-isoprenylcysteine O-methyltransferase B（ICMTB） |
|  |  | chr19_11081533 |  |  |
|  |  | chr19_11081535 |  |  |
|  |  | chr19_11079056 |  |  |
|  |  | chr19_11066561 |  |  |
|  |  | chr19_11081719 |  |  |
|  |  | chr19_11083446 |  |  |
|  |  | chr19_11075153 |  |  |
|  |  | chr19_11078705 |  |  |
|  |  | chr19_11079794 |  |  |
|  |  | chr19_11074046 |  |  |
|  |  | chr19_11067137 |  |  |
|  |  | chr19_11073782 |  |  |
|  |  | chr19_11078147 |  |  |
|  |  | chr19_11065612 |  |  |
|  |  | chr19_11061515 |  |  |
|  |  | chr19_11068309 |  |  |
|  |  | chr19_11083587 |  |  |
|  |  | chr19_11068771 |  |  |
|  |  | chr19_11079753 |  |  |
|  |  | chr19_11066626 |  |  |
|  |  | chr19_11081667 |  |  |
|  |  | chr19_11081677 |  |  |
|  |  | chr19_11085450 |  |  |
|  |  | chr19_11085342 |  |  |
|  |  | chr19_11068733 |  |  |
|  |  | chr19_11075224 |  |  |
|  |  | chr19_11073426 |  |  |
|  |  | chr19_11086619 |  |  |
|  |  | chr19_11076669 |  |  |
|  |  | chr19_11076713 |  |  |
|  |  | chr19_11073618 |  |  |
|  |  | chr19_11075245 |  |  |
|  |  | chr19_11074271 |  |  |
|  |  | chr19_11081786 |  |  |
|  |  | chr19_11073479 |  |  |
|  |  | chr19_11066476 |  |  |
|  |  | chr19_11081142 |  |  |
|  |  | chr6_32508113 |  |  |
|  |  | chr19_11081784 |  |  |
|  |  | chr19_11077798 |  |  |
|  |  | chr19_11082222 |  |  |
|  |  | chr6_32507414 |  |  |
|  |  | chr19_11083869 |  |  |
|  |  | chr19_11076367 |  |  |
|  |  | chr19_11081954 |  |  |
|  |  | chr6_32506644 |  |  |
|  |  | chr19_24605141 |  |  |
|  |  | chr19_11083366 |  |  |
|  |  | chr19_11085057 |  |  |
|  |  | chr19_11085175 |  |  |
|  |  | chr19_11074943 |  |  |
|  |  | chr6_32506685 |  |  |
|  |  | chr6_32505897 |  |  |
|  |  | chr19_11079173 |  |  |
|  |  | chr19_24601467 |  |  |
|  |  | chr19_11086605 |  |  |
|  |  | chr6_32506892 |  |  |
|  |  | chr19_11074113 |  |  |
|  |  | chr19_11082893 |  |  |
|  |  | chr6_32506962 |  |  |
|  |  | chr19_11080000 |  |  |
|  |  | chr4_351031 |  |  |
|  |  | chr19_11076585 |  |  |
|  |  | chr19_24606421 |  |  |
|  |  | chr19_11076797 |  |  |
|  |  | chr19_11083053 |  |  |
|  |  | chr19_11074278 |  |  |
|  |  | chr6_32506436 |  |  |
|  |  | chr4_380330 |  |  |
|  |  | chr4_380336 |  |  |
|  |  | chr19_11077255 |  |  |
|  |  | chr19_11068066 |  |  |
|  |  | chr19_24608985 |  |  |
|  |  | chr4_380335 |  |  |
|  |  | chr19_11075962 |  |  |
|  |  | chr19_24605318 |  |  |
|  |  | chr19_11081295 |  |  |
|  |  | chr6_29034217 |  |  |
|  |  | chr19_24597096 |  |  |
|  |  | chr19_11079655 |  |  |
|  |  | chr19_11083526 |  |  |
|  |  | chr19_11083525 |  |  |
|  |  | chr5_2217466 |  |  |
|  |  | chr19_11083150 |  |  |
|  |  | chr4_188798 |  |  |
|  |  | chr4_122295 |  |  |
|  |  | chr6_32506262 |  |  |
|  |  | chr19_24597105 |  |  |
|  |  |  |  |  |
|  | BD | chr19_12496876 | *evm.model.Contig139.72* | unnamed protein product |
|  |  | chr19_12432456 | *evm.model.Contig139.75* | unnamed protein product |
|  |  | chr19_12634808 | *evm.model.Contig139.76* | unnamed protein product |
|  |  | chr19_12637900 | *evm.model.Contig139.77.1* | vacuolar fusion protein CCZ1 homolog B-like isoform X4 |
|  |  | chr19_12268119 | *evm.model.Contig139.84* | unnamed protein product |
|  |  | chr19_12465737 | *evm.model.Contig139.85* | unnamed protein product |
|  |  | chr19_12728802 | *evm.model.Contig139.86* | unnamed protein product |
|  |  | chr03_9878370 | *evm.model.Contig139.87* | PREDICTED: PHD finger protein MALE MEIOCYTE DEATH 1-like |
|  |  | chr19_12579563 | *evm.model.Contig139.88* | putative late blight resistance protein homolog R1A-10 |
|  |  | chr19_12518192 | *evm.model.Contig139.89* | putative late blight resistance protein homolog R1A-10 |
|  |  | chr19_12521701 | *evm.model.Contig139.90* | putative late blight resistance protein homolog R1A-10 |
|  |  | chr19_12548824 | *evm.model.Contig139.91* | unnamed protein product |
|  |  | chr19_12579769 | *evm.model.Contig139.93* | unnamed protein product |
|  |  | chr19_12607464 | *evm.model.Contig139.94* | aminoacylase-1-like |
|  |  | chr19_12672888 | *evm.model.Contig139.95* | miraculin-like |
|  |  | chr19_12732578 | *evm.model.Contig139.96* | protein RGF1 INDUCIBLE TRANSCRIPTION FACTOR 1-like |
|  |  | chr19_12758085 | *evm.model.Contig418.13* | unnamed protein product |
|  |  | chr03_9907678 | *evm.model.Contig418.12* | unnamed protein product |
|  |  | chr19_12752839 | *evm.model.Contig418.15.1* | unnamed protein product |
|  |  | chr03_9877077 |  |  |
|  |  | chr19_12442083 |  |  |
|  |  | chr19_12565939 |  |  |
|  |  | chr19_12258965 |  |  |
|  |  | chr19_12453769 |  |  |
|  |  | chr19_12641471 |  |  |
|  |  | chr19_12641477 |  |  |
|  |  | chr19_12221520 |  |  |
|  |  | chr19_12442717 |  |  |
|  |  | chr19_12550101 |  |  |
|  |  | chr19_12594818 |  |  |
|  |  | chr19_12634482 |  |  |
|  |  | chr19_12638682 |  |  |
|  |  | chr19_12655612 |  |  |
|  |  | chr19_12689253 |  |  |
|  |  | chr19_12736182 |  |  |
|  |  | chr19_12737524 |  |  |
|  |  | chr19_12767901 |  |  |
|  |  | chr19_12783132 |  |  |
|  |  | chr19_12789316 |  |  |
|  |  | chr03_9908152 |  |  |
|  |  | chr19_12594443 |  |  |
|  |  | chr19_12594444 |  |  |
|  |  | chr19_12781364 |  |  |
|  |  | chr19_12565190 |  |  |
|  |  | chr19_12440346 |  |  |
|  |  | chr03_9907443 |  |  |
|  |  | chr19_12425059 |  |  |
|  |  | chr19_12407403 |  |  |
|  |  | chr19_12691235 |  |  |
|  |  | chr03_9881508 |  |  |
|  |  | chr19_12431540 |  |  |
|  |  | chr19_12799009 |  |  |
|  |  | chr19_12513346 |  |  |
|  |  | chr03_9877204 |  |  |
|  |  | chr19_12085710 |  |  |
|  |  | chr19_12481713 |  |  |
|  |  | chr03_9881935 |  |  |
|  |  | chr19_12240158 |  |  |
|  |  | chr03_9880550 |  |  |
|  |  | chr19_12496876 |  |  |
|  |  | chr19_12432456 |  |  |
|  |  | chr19_12634808 |  |  |
|  |  | chr19_12637900 |  |  |
|  |  | chr19_12268119 |  |  |
|  |  | chr19_12465737 |  |  |
|  |  | chr19_12728802 |  |  |
|  |  | chr03_9878370 |  |  |
|  |  | chr19_12579563 |  |  |
|  |  | chr19_12518192 |  |  |
|  |  | chr19_12521701 |  |  |
|  |  | chr19_12548824 |  |  |
|  |  | chr19_12579769 |  |  |
|  |  | chr19_12607464 |  |  |
|  |  | chr19_12672888 |  |  |
|  |  | chr19_12732578 |  |  |
|  |  | chr19_12758085 |  |  |
|  |  | chr03_9907678 |  |  |
|  |  | chr19_12752839 |  |  |
|  |  | chr03_9877077 |  |  |
|  |  |  |  |  |
|  | VL/  VD | chr01_33454649 | *evm.model.Contig81.326* | uncharacterized protein |
|  |  | chr15_16835862 | *evm.model.Contig81.328* | uncharacterized protein |
|  |  | chr15_16823696 | *evm.model.Contig81.329* | uncharacterized protein |
|  |  | chr15_16942644 | *evm.model.Contig81.334.1* | APETALA2-like protein 3 isoform X2 |
|  |  | chr15_16914008 | *evm.model.Contig81.332* | CCR4-NOT transcription complex subunit 4-like isoform X1 |
|  |  | chr15_16864218 | *evm.model.Contig81.325.1* | uncharacterized protein |
|  |  | chr15_16976841 | *evm.model.Contig81.330* | patatin-like protein 2 |
|  |  | chr15_16923695 | *evm.model.Contig81.324* | probable RNA-binding protein ARP1 |
|  |  | chr15_16663649 | *evm.model.Contig81.333* | unnamed protein product |
|  |  | chr15_16901901 | *evm.model.Contig81.327* | unnamed protein product |
|  |  | chr15_16921376 | *evm.model.Contig39.53.2* | salicylic acid-binding protein 2-like |
|  |  | chr15_16950365 | *evm.model.Contig39.55* | salicylic acid-binding protein 2-like |
|  |  |  | *evm.model.Contig39.54* | salicylic acid-binding protein 2-like |
|  |  |  | *evm.model.Contig39.57* | salicylic acid-binding protein 2-like |
|  |  |  | *evm.model.Contig39.59* | hyoscyamine 6-dioxygenase |
|  |  |  | *evm.model.Contig298.88* | protein RGF1 INDUCIBLE TRANSCRIPTION FACTOR 1-like |
|  |  |  | *evm.model.Contig298.85.1* | tRNA threonylcarbamoyladenosine dehydratase-like isoform X2 |
|  |  |  | *evm.model.Contig298.93* | ethylene-responsive transcription factor 1B-like |
|  |  |  | *evm.model.Contig298.92* | ethylene-responsive transcription factor 1B-like |
|  |  |  | *evm.model.Contig298.95* | ethylene-responsive transcription factor 1B-like |
|  |  |  | *evm.model.Contig298.94* | ethylene-responsive transcription factor 1B-like |
|  |  |  | *evm.model.Contig298.91* | unnamed protein product |
|  |  |  | *evm.model.Contig298.86* | potassium channel SKOR isoform X1 |
|  |  |  | *evm.model.Contig298.87* | unnamed protein product |
|  |  |  | *evm.model.Contig298.89* | probable methyltransferase At1g29790 |
|  |  |  |  |  |
| 2^nd^ | FD | chr20_17634027 | *evm.model.Contig171.188* | unnamed protein product |
|  |  | chr11_13287624 | *evm.model.Contig171.189* | transcriptional corepressor SEUSS-like |
|  |  | chr19_18231979 | *evm.model.Contig171.190* | transcriptional corepressor SEUSS-like |
|  |  | chr11_14195852 | *evm.model.Contig171.191* | Small RNA degrading nuclease 5 |
|  |  |  | *evm.model.Contig171.192* | small RNA degrading nuclease 5 isoform X2 |
|  |  |  | *evm.model.Contig171.193* | unnamed protein product |
|  |  |  | *evm.model.Contig171.194* | scarecrow-like protein 1 |
|  |  |  | *evm.model.Contig171.195* | unnamed protein product |
|  |  |  | *evm.model.Contig268.9* | protein BASIC PENTACYSTEINE1-like |
|  |  |  | *evm.model.Contig330.70* | probable xyloglucan glycosyltransferase 5 |
|  |  |  |  |  |
|  | VD | chr17_3824776 | *evm.model.Contig81.140* | silencing protein 10 isoform X1 |
|  |  | chr17_3825173 | *evm.model.Contig81.141* | silencing protein 10 isoform X1 |
|  |  |  | *evm.model.Contig81.142* | *ALA-interacting subunit 3-like* |
|  |  |  | *evm.model.Contig81.166* | *uncharacterized protein* |
|  |  |  |  |  |
|  | BD | chr01_31547953 | *evm.model.Contig5.176* | *uncharacterized protein* |
|  |  |  | *evm.model.Contig5.177* | *unnamed protein product* |
|  |  |  | *evm.model.Contig5.178* | *uncharacterized protein* |
|  |  |  | *evm.model.Contig5.179* | *uncharacterized protein* |
|  |  |  | *evm.model.Contig5.180* | *unnamed protein product* |
|  |  |  | *evm.model.Contig5.181_evm.model.Contig5.182* | *galactan beta-1,4-galactosyltransferase GALS3-like* |
|  |  |  | *evm.model.Contig5.185* | *uncharacterized protein* |
|  |  |  | *evm.model.Contig5.186* | *ycf3-interacting protein 1* |
|  |  |  |  |  |
|  | FL/  FD |  | *evm.model.Contig583.11* | *unnamed protein product* |
|  |  |  | *evm.model.Contig583.12* | *protein SMALL AUXIN UP-REGULATED RNA 51-like* |
|  |  |  | *evm.model.Contig583.13* | *UDP-glycosyltransferase 86A2-like* |
|  |  |  | *evm.model.Contig583.14* | *ABC transporter G family member 3* |
|  |  |  | *evm.model.Contig583.15* | *unnamed protein product* |
|  |  |  | *evm.model.Contig583.16* | *unnamed protein product* |
|  |  |  | *evm.model.Contig583.17* | *uncharacterized protein* |
|  |  |  | *evm.model.Contig583.18* | *unnamed protein product* |
|  |  |  | *evm.model.Contig583.19* | *unnamed protein product* |
|  |  |  | *evm.model.Contig583.20* | *High-affinity nitrate transporter 2.1* |

**Table S14** Comparison of gene-family members relevant to lignin biosynthesis among *A. thaliana*, *N. cadamba* and *N. macrophylla*

| Gene Family | Number of  *A. thaliana* | *N. cadamba* | *N. macrophylla* |
| --- | --- | --- | --- |
| PAL | 4 | 7 | 8 |
| C4H | 1 | 3 | 3 |
| 4CL | 4 | 3 | 4 |
| COMT | 1 | 1 | 2 |
| F5H | 1 | 5 | 5 |
| CCoAOMT | 2 | 2 | 2 |
| CCR | 2 | 2 | 2 |
| CAD  CSE | 9  1 | 10  2 | 14  2 |
| HCT  C3H | 1  1 | 4  2 | 4  3 |

**Table S15** Detection of selective pressure on gene families for lignin biosynthesis in *Neolamarckia*

|  | Gene Family* | Homologous gene pairs | Ka/Ks  (mean ± SD) | Ka/Ks range  [min, max] |
| --- | --- | --- | --- | --- |
| Intraspecific | At-At PAL | 2 | 0.064±0.019 | [0.051, 0.078] |
|  | Nc-Nc PAL | 10 | 0.579±0.214 | [0.199, 0.840] |
|  | Nm-Nm PAL | 15 | 0.029±0.018 | [0.019, 0.078] |
|  | At-At C4H | - | - | - |
|  | Nc-Nc C4H | 1 | 0.538 | [0.538, 0.538] |
|  | Nm-Nm C4H | 2 | 0.254±0.135 | [0.031, 0.223] |
|  | At-At 4CL | 1 | 0.073±0 | [0.073, 0.073] |
|  | Nc-Nc 4CL | - | - | - |
|  | Nm-Nm 4CL | 1 | 0.087±0 | [0.087, 0.087] |
|  | At-At HCT | - | - | - |
|  | Nc-Nc HCT | 3 | 0.420±0.107 | [0.300, 0.505] |
|  | Nm-Nm HCT | 6 | 0.063±0.008 | [0.056, 0.075] |
|  | At-At C3H | - | - | - |
|  | Nc-Nc C3H | - | - | - |
|  | Nm-Nm C3H | 2 | 0.195±0.177 | [0.07, 0.320] |
|  | At-At CSE | - | - | - |
|  | Nc-Nc CSE | 1 | 0.388 | [0.388, 0.388] |
|  | Nm-Nm CSE | 1 | 0.093 | [0.093, 0.093] |
|  | At-At CCoAOMT | - | - | - |
|  | Nc-Nc CCoAOMT | 1 | 0.402 | [0.402, 0.402] |
|  | Nm-Nm CCoAOMT | 1 | 0.057 | [0.057, 0.057] |
|  | At-At CCR | 1 | 0.080 | [0.080, 0.080] |
|  | Nc-Nc CCR | 1 | 0.453 | [0.453, 0.453] |
|  | Nm-Nm CCR | 1 | 0.084 | [0.084,0.084] |
|  | At-At CAD | 1 | 0.108 | [0.108, 0.108] |
|  | Nc-Nc CAD | 8 | 0.486±0.075 | [0.402, 0.599] |
|  | Nm-Nm CAD | 2 | 0.084±0.008 | [0.078, 0.090] |
|  | At-At COMT | - | - | - |
|  | Nc-Nc COMT | 0 | - | - |
|  | Nm-Nm COMT | 1 | 0.119 | [0.119, 0.119] |
|  | At-At F5H | - | - | - |
|  | Nc-Nc F5H | 1 | 0.257 | [0.257, 0.257] |
|  | Nm-Nm F5H | 1 | 0.082 | [0.082, 0.082] |
| Interspecific | Nc-At PAL | 8 | 0.434±0.124 | [0.225, 0.568] |
|  | Nm-At PAL | 10 | 0.032±0.006 | [0.026, 0.043] |
|  | Nm-Nc PAL | 29 | 0.398±0.006 | [0.248, 0.558] |
|  | Nc-At C4H | 4 | 0.505±0.006 | [0.318, 0.699] |
|  | Nm-At C4H | 4 | 0.145±0.148 | [0.016, 0.278] |
|  | Nm-Nc C4H | 6 | 0.471±0.131 | [0.297, 0.615] |
|  | Nc-At 4CL | - | - | - |
|  | Nm-At 4CL | - | - | - |
|  | Nm-Nc 4CL | 4 | 0.608±0.146 | [0.398, 0.724] |
|  | Nc-At HCT | 1 | 0.687 | [0.687, 0.687] |
|  | Nm-At HCT | - | - | - |
|  | Nm-Nc HCT | 10 | 0.545±0.116 | 0.694 |
|  | Nc-At C3H | 1 | 0.600 | [0.600, 0.600] |
|  | Nm-At C3H | 2 | 0.045±0.002 | [0.042, 0.046] |
|  | Nm-Nc C3H | 2 | 0.515±0.025 | [0.497, 0.532] |
|  | Nc-At CSE | 2 | 0.516±0.067 | [0.468, 0.563] |
|  | Nm-At CSE | 2 | 0.033±0.0001 | [o.0330, 0.0332] |
|  | Nm-Nc CSE | 4 | 0.440±0.048 | [0.371, 0.482] |
|  | Nc-At CCoAOMT | 2 | 0.496±0.115 | [0.415, 0.577] |
|  | Nm-At CCoAOMT | 4 | 0.050±0.042 | [0.013, 0.094] |
|  | Nm-Nc CCoAOMT | 4 | 0.463±0.098 | [0.358, 0.547] |
|  | Nc-At CCR | 4 | 0.668±0.093 | [0.548, 0.747] |
|  | Nm-At CCR | 4 | 0.046±0.007 | [0.037, 0.054] |
|  | Nm-Nc CCR |  | 0.693±0.178 | [0.594, 0.960] |
|  | Nc-At CAD | 5 | 0.343±0.283 | [0.048, 0.688] |
|  | Nm-At CAD |  | 0.055±0.027 | [0.036, 0.101] |
|  | Nm-Nc CAD | 12 | 0.550±0.100 | [0.436, 0.805] |
|  | Nc-At COMT | 1 | 0.788 | [0.788, 0.788] |
|  | Nm-At COMT | 1 | 0.032 | [0.032,0.032] |
|  | Nm-Nc COMT | 2 | 0.586±0.051 | [0.550, 0.622] |
|  | Nc-At F5H | 2 | 0.453±0.157 | [0.342, 0.564] |
|  | Nm-At F5H | 2 | 0.044±0.0004 | [0.043, 0.044] |
|  | Nm-Nc F5H | 5 | 0.426±0.111 | [0.323, 0.577] |

*: Nm: *N. macrophylla*; Nc: *N. cadamba*; At: *A. thaliana*

**Table S16** Comparison of repeat contents in *Neolamarckia macrophylla* versus *N. cadamba* genome.

|  | *N. macrophylla* | | *N. cadamba* | |
| --- | --- | --- | --- | --- |
|  | Length(bp) | % in genome | Length(bp) | % in genome |
| DNA transposon | 37,310,484 | 6.23 | 21,529,812 | 2.89 |
| LINE | 6,870,787 | 1.15 | 13,689,416 | 1.84 |
| SINE | 461,044 | 0.08 | 15,534 | 0.00 |
| LTR | 199,516,424 | 33.34 | 344,987,362 | 46.34 |
| Unknown | 2,319 | 0.00 | 164 | 0.00 |
| Total | 244,161,058 | 40.8 | 399,833,705 | 53.71 |

**Figure S1.** K-mer frequency distribution in the *N. macrophylla* genome. The horizontal axis represents the depth of the K-mer, and the vertical axis represents the frequency of the K-mer at a given depth.

**Figure S2.** GO enrichment analysis of species-specific gene families in *N. macrophylla*

**Figure S3.** KEGG analysis of species-specific gene families in *N. macrophylla*

**Figure S4.** GO enrichment analysis of expanded gene families in *N. macrophylla*

**Figure S5**. KEGG analysis of expanded gene families in *N. macrophylla*

**Figure S6**. Distribution of p-values from Hardy-Weinberg equilibrium (HWE) testing in the two samples of *N. cadamba*

**Figure S7.** Distributions of nucleotide diversity (π) per 1-kb window size in two samples of *N. cadamba*

**Figure S8.** Quantile-quantile plots for GWAS on wood traits of *N. cadamba*. (A) FL; (B) FD; (C) VL; (D) VD; (E) BD; (F) VL/VD; (G) FD; (H) VD (I) BD; (J) FL/FD. Figures (A) -(F) refer to GWAS in the first sample, whereas (G)-(J) refer to the results in the second sample.
